# The Impact of N/O-Functional Groups on the Sorption Capabilities of Activated Carbons Derived from Furfuryl Alcohol

**DOI:** 10.3390/molecules29050987

**Published:** 2024-02-24

**Authors:** Agnieszka Kałamaga, Rafał J. Wróbel

**Affiliations:** Department of Catalytic and Sorbent Materials Engineering, Faculty of Chemical Technology and Engineering, West Pomeranian University of Technology, Piastów 17 Ave., 70-310 Szczecin, Poland

**Keywords:** activated carbons, nitrogen and oxygen modification, adsorption, carbon dioxide, ethylene, sorption applications

## Abstract

This work describes the effect of nitrogen and oxygen functional groups on the sorption properties of activated carbons produced from furfuryl alcohol. The poly(furfuryl) alcohol underwent carbonization in nitrogen, ammonia, and ammonia and air (in a 3:2 proportion) atmospheres at 600 °C for 4 h. The resulting materials were subsequently activated in a carbon dioxide atmosphere for 1 h at temperatures of 700 °C, 800 °C, 900 °C, and 1000 °C. The X-ray photoelectron spectroscopy (XPS) findings suggest that ammoxidation is superior to amination in terms of nitrogen doping. The maximum nitrogen concentration achieved after ammoxidation was 25 at.%, which decreased to 4 at.% after activation. Additionally, it was observed that oxygen functional groups have a greater impact on porous structure development compared to nitrogen functional groups. The materials activated through carbonization under an ammonia/air atmosphere attained the highest oxygen concentration of roughly 19 at.% as confirmed by XPS. The materials were evaluated for their sorption capacities for carbon dioxide and ethylene, which were 2.2 mmol/g and 2.9 mmol/g, respectively, at 30 °C.

## 1. Introduction

Since the latter half of the 20th century, there has been a global rise in the average surface temperature of the Earth. This has been attributed to an increase in the greenhouse effect caused by excessive carbon dioxide emissions, primarily from the combustion of fossil fuels. Based on years of observation, it has been noted that the intensified greenhouse effect has numerous adverse effects. Processes such as absorption, adsorption, cryogenic separation, and membrane separation are used to remove carbon dioxide from gases. The method chosen for flue gases depends on their parameters and the associated energy production costs.

Activated carbons are commonly used in adsorption processes to reduce carbon dioxide emissions into the atmosphere due to their low production costs, availability of raw materials, and ease of modification. The adsorption processes can be classified into four types: temperature swing adsorption, pressure swing adsorption, vacuum swing adsorption, and pressure–temperature swing adsorption [1].

Activated carbons have an interesting application in the sorption of ethylene resulting from the ripening of vegetables and fruits. This is particularly relevant for climacteric produce, such as tomatoes, apples, pears, peaches, avocados, and bananas. The process of climacteric ripening in these produce items is linked to changes in the concentration of ethylene, which is the main hormone regulating the biochemical processes occurring in their structures. In plant cells, ethane is produced through the transformation of methionine. Additionally, receptors in plant tissues detect airborne ethylene. In both cases, high concentrations of ethylene lead to biochemical changes that accelerate the ripening of climacteric fruits and vegetables. In summary, the adsorption of ethylene is crucial to preserve the quality of products. Therefore, it is important to remove ethylene, especially during food storage [2,3].

For numerous years, activated carbons have also been widely used in a variety of processes, which include water treatment [4,5,6,7,8,9], air purification [10,11,12,13], gas phase applications [14,15], medical and pharmaceutical applications [7,16,17,18], food and beverage industry applications [19], energy storage and electrochemical applications [20,21,22,23,24,25], and catalysis and chemical processes [26,27,28].

The chemical composition of the surface and the porous structure are the main influencing factors regarding the application of a material in a specific field [12,29,30,31,32]. Recently, many researchers have focused on understanding the effects of oxygen and nitrogen heteroatoms on the properties of carbon materials. Carbonaceous materials comprising nitrogen and oxygen heteroatoms in their composition can be classified into two categories: materials derived from raw materials containing nitrogen and oxygen and materials doped with them during synthesis [33,34]. Raw materials used to produce activated carbons, which have high concentrations of nitrogen and oxygen, include polymers such as polyamide [35], polyacrylonitrile [36,37,38], or biomass, including, for example, coffee beans [33] or nut shells [6,39,40,41,42]. The main substances used for the nitrogen and oxygen doping of activated carbons are included in the second group and comprise urea [34,43,44,45], melamine [46,47,48], PEI [49,50], nitric acid [51,52], acetic acid [53], ozone [54], and metal oxides or salts [55,56,57,58].

It has been observed that the introduction of nitrogen-containing functional groups leads to an increase in basic sites [59]. These sites provide the appropriate binding energy for carbon dioxide molecules. Nitrogen-containing functional groups can take on various forms, including pyrydinic, pyrrolic, pyridonic, quaternary, amine, nitro, and nitroso types, as reported in the literature. All of these elements, except for quaternary nitrogen, occur at the edge of the graphitic plane. Furthermore, it is important to note that pyrrolic and pyridonic nitrogen cannot be distinguished using XPS and are therefore analyzed together. Sun et al. observed that CO_2_ binds more strongly to pyrydinic and pyrrolic nitrogen than to quaternary nitrogen due to the formation of electrostatic bonds with the former and dispersion bonds with the latter [60]. Ma and co-workers reported that the strongest contributor to CO_2_ adsorption was the Lewis acid–base interactions between CO_2_ and pyrydinic nitrogen, with a binding energy of 21.26 kJ mol^−1^ [61]. This was followed by pyrrolic nitrogen, which had binding energies of 18.60 and 10.82 kJ mol^−1^, and amine nitrogen, which had binding energies of 16.45 and 5.72 kJ mol^−1^ and demonstrates two configurations of CO_2_ adsorption. Pyrrolic nitrogen and amine groups promote both Lewis acid–base and hydrogen bonding interactions between nitrogen and hydrogen atoms and CO_2_. Additionally, it was reported that hydrogen bonding interactions played a dominant role [62]. Pyridine nitrogen affects CO_2_ adsorption through exceptional hydrogen bonding interactions with CO_2_ [61].

On the surface of activated carbons, oxygen functional groups also play an equally important role [59]. These oxygen groups are important because they contribute to two properties of carbon adsorbents: hydrophobicity/hydrophilicity and acidity/basicity. A carbon surface lacking oxygen functional groups is hydrophobic. Its hydrophilicity is increased by the addition of oxygen functional groups. In the case of the adsorption of CO_2_, there is an increased competition for active sites with the water vapor of flue gas. The functional groups of oxygen can be classified as acidic, basic, or neutral. The acidic behavior is associated with surface complexes or functionalities containing oxygen, such as carboxyls, lactones, and phenols. On the other hand, pyrones, chromenes, ethers, carbonyls, and hydroxyl groups are responsible for the basic properties of a carbon surface. In addition, oxygen functional groups like hydroxyl, carbonyl, or ether contain an electron donor atom that may interact electrostatically with CO_2_. Reports in the literature suggest that the alkalinity of the hydroxyl group is a positive factor for CO_2_ adsorption [63]. The high electron density of both hydroxyl and carbonyl groups also affects CO_2_ adsorption. The interaction between the hydroxyl group and CO_2_ is characterized by high electrostatic potential [61]. Additionally, hydrogen bonds can form between hydroxyl and CO_2_, which contribute to the adsorption process. Plaza et al. observed Lewis’ strong acid–base interactions between the CO_2_ and carboxyl groups [64].

Many papers aim to explain the individual effects of functional groups that contain nitrogen and oxygen, as well as their synergistic effects. Several researchers have concluded that nitrogen and oxygen heteroatoms have effects on the properties of activated carbons.

Zhang et al. produced carbon materials using expired coffee for high-capacity supercapacitors by subjecting it to carbonization between 600 °C and 800 °C in a water vapor atmosphere. The nitrogen and oxygen concentrations reached their maximum values during carbonization at 600 °C, measuring 4.5% and 12.0%, respectively. However, elevating the carbonization temperature to 800 °C resulted in declines in the nitrogen and oxygen concentrations to 3.1% and 8.6%, respectively. Moreover, an increase in the water vapor carbonization temperature was found to facilitate the deep oxygenation of the carbon skeleton due to the rise in the concentration of carboxylic groups [65].

Zapata-Benabithe and co-authors produced carbon materials using organic aerogels (resorcinol and pyrocatechol) via chemical activation with potassium hydroxide. They subjected the aerogels to pyrolysis at 300 °C for 3 h before heating them at 840 °C for 2 h in a nitrogen atmosphere. Ammonium peroxysulphate and melamine were utilized as sources of oxygen and nitrogen, respectively. A more developed porous structure was achieved for carbons produced utilizing resorcinol in contrast to pyrocatechol. The highest pore volume was 0.8 cm^3^/g. The researchers demonstrated that both oxygen and nitrogen doping lead to a decrease in the porosity of the obtained materials. In addition, it was also observed that the decrease in porosity was greater after modification with oxygen compounds than with nitrogen compounds. The reduction in porosity was found to be closely related to the blocking of the surface by oxygen groups or the oxidation of micro- and mesopore walls. The highest oxygen concentration was achieved by the resorcinol series (17.2 wt.%). The nitrogen concentration was the same for both series (1.4 wt.%) [66].

Luo et al. produced activated carbons from Chinese fir bark using potassium hydroxide activation at a temperature of 700 °C with a mass ratio of 1.0:0.5–6.0. The findings indicate that the maximum nitrogen and oxygen contents were 1.5 wt.% and 7.4 wt.%, respectively. The researchers noted that the adsorption capacity of carbon dioxide decreased with the rises in nitrogen and oxygen contents. However, separating the interrelated effects of porosity and chemical composition is difficult because the concentration of heteroatoms and the structures of the pores vary with weight ratios [67].

Plaza et al. conducted a 2 h ammoxidation process at 200 °C and 300 °C using a gas mixture of ammonia and air at a ratio of 1:2 on two types of commercial activated carbons. The maximum nitrogen and oxygen concentrations achieved were 9.2 wt.% and 13.8 wt.%, respectively. The researchers noted that the ammoxidation process affected carbon dioxide adsorption in two distinct ways. On the one hand, ammoxidation decreases the porous volume of untreated carbon, resulting in decreased carbon dioxide adsorption at room temperature. Furthermore, modified materials show slightly higher carbon dioxide adsorption at 100 °C compared to non-modified carbon, which is likely due to changes in surface chemistry that have a greater impact at higher temperatures. In the case of the second series, ammoxidation does not have an effect on the porous structure. However, it was observed that the adsorption of carbon dioxide showed a slight increase. The authors hypothesize that this may be attributed to the introduction of nitrogen functionalities through doping [68].

Wang and co-workers carried out KOH activation using commercial carbons to separate ethane/ethylene. The 1 h activation processes were conducted at 700 °C, 800 °C, and 900 °C. The concentration of oxygen was the highest in the unactivated carbon (16.9 at.%), and this was reduced to 7.4 at.% with an increase in the activation temperature. The content of nitrogen varied between 0.7 at.% and 2.0 at.% throughout the processes. The researchers noted that KOH activation only acts as an activator and creates pores but does not aid in the enhancement of functional sites [69].

In our recent study, the objective was to examine the effects of ammonium nitrate and ammonium perchlorate on the porous structure of activated carbons specifically for carbon dioxide and ethylene adsorption. The ammonium-perchlorate-modified material obtained the highest oxygen concentration of 13.0 at.%. The nitrogen content was between 2.0 at.% and 4.0 at.% and stayed comparable in both series of materials. Activated carbons obtained from the study exhibited superior sorption properties for ethylene compared to carbon dioxide. At 30 °C, the maximum sorption capacities for ethylene and carbon dioxide were 3.4 mmol/g and 2.1 mmol/g, respectively. Furthermore, the present study demonstrates that the addition of ammonium nitrate to the precursor composition enables a reduction in the activation time while attaining comparable sorption capacities at both 1 h and 2 h activation times [70].

The purpose of this research is to investigate the influences of nitrogen and oxygen functional groups on the sorption and structural properties of furfuryl-alcohol-derived activated carbons. In order to avoid the influence of mineral matter on activated carbons’ properties, carbonaceous materials were prepared from a high-purity precursor without nitrogen in the chemical composition. The nitrogen doping was carried out during carbonization processes by amination under an ammonia atmosphere and by ammoxidation under an ammonia/air atmosphere. The second objective of this work is to differentiate the influences of nitrogen and oxygen functional groups on the surfaces of carbons on gas adsorption from that of the porous structure.

## 2. Results and Discussion

The yield of activated carbon is usually defined as the weight of final activated carbon produced after activation and divided by the mass of the raw material.

In Table 1, the carbonization process yields under nitrogen, ammonia, and ammonia/air atmospheres are presented.

The material yield after carbonization is significantly affected by the carbonization atmosphere, as shown in Table 1. The carbonization processes carried out under nitrogen (PFA-N_2_ series), ammonia (PFA-NH_3_ series), and an ammonia/air mixture (PFA-NH_3_/air series) resulted in materials with different chemical compositions, which was confirmed by the XPS spectra. The XPS spectra were used to estimate the concentrations of carbon, oxygen, and nitrogen over the surfaces of carbonaceous materials.

Significant variations in surface chemistry were observed in the materials following the carbonization processes. The results are presented in Table 2. The XPS spectra of the precursor and carbonized materials can be found in the Supplementary Materials (Figure S1). SEM images of the precursor and post-carbonization materials are provided in the Supplementary Materials (Figure S2).

A comparison of the surface chemistry of precursor and carbonized materials shows that the ammoxidation process resulted in the highest nitrogen and oxygen concentrations. In contrast, amination resulted in only 6.0 at.% of nitrogen and 3.0 at.% of oxygen in the chemical composition. After carbonization in a nitrogen atmosphere, the oxygen concentration was 5.0 at.% with a nitrogen content below the detection limit.

Carbonization under nitrogen is a decomposition process involving aliphatic acids, carbonyl compounds, and alcohols, among other materials. The reaction also leads to the removal of elements such as nitrogen, hydrogen, and oxygen, while increasing the ratio of elemental carbon to hydrogen. Carbonization under an ammonia atmosphere results in the formation of nitrogen-containing functional groups through the reaction of ammonia with oxygen functional groups on the precursor surface. Additionally, carbonization in an ammonia/air atmosphere leads to the simultaneous oxidation and amination of the material, resulting in a higher concentration of nitrogen groups on the carbon surface compared to carbonization under an ammonia atmosphere. It can be inferred that carbonization in a nitrogen atmosphere removes oxygen groups. When carbonized in ammonia and ammonia/air atmospheres, the oxygen groups on the precursor’s surface do not become removed, but instead react with ammonia. Thus, the PFA-N_2_ material has the lowest material yield, while the PFA-NH_3_/air material has the highest.

In order to achieve high sorption performance, the carbonized materials were subjected to physical activation in a CO_2_ atmosphere. During this process, a substantial part of the material is transformed into gaseous products, resulting in the enhancement of the porous structure. However, the carbon gasification rate is dependent on the oxygen and nitrogen contents.

The activation process yields under CO_2_ activation are provided in Table 3.

Table 3 shows that the highest activation yield was achieved at 700 °C. As the activation temperature increased, the yield decreased. For the temperature range of 700 °C to 900 °C, the material yield was similar for each of the material series. Significant differences between the material yields for each series were observed only after activation at 1000 °C. At this temperature, sample PFA-N_2_-1000 yielded the most material (70%), followed by sample PFA-NH_3_-1000 (62%). On the other hand, the PFA-NH_3_/air-1000 sample yielded the lowest amount of material (55%).

**Figure 1:**
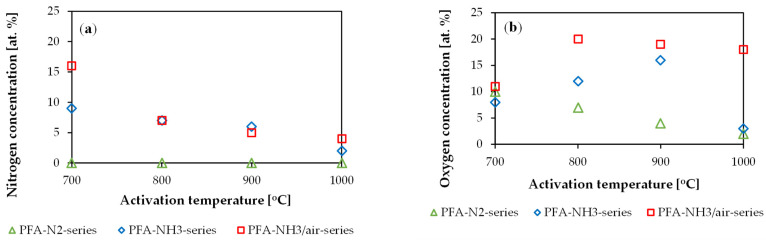
(**a**) Nitrogen and (**b**) oxygen concentrations on the surfaces of carbons after CO_2_ activation processes from 700 °C to 1000 °C under CO_2_ atmosphere.

The nitrogen and oxygen surface concentrations of the materials vary during the activation processes and are illustrated in Figure 1.

Figure 1 shows that there is a decrease in the nitrogen concentration as the temperature rises from 700 °C to 1000 °C. This may indicate that nitrogen-doped carbon preferentially reacts with CO_2_ during the activation process. The PFA-NH_3_/air series has a higher nitrogen content than the PFA-NH_3_ series following activation at 700 °C. Nevertheless, activation processes at 800 °C, 900 °C, and 1000 °C resulted in materials with comparable nitrogen contents.

The oxygen concentration is comparable for materials activated at 700 °C. When examining the PFA-NH_3_/air-series, the increase in the temperature from 800 °C to 1000 °C caused an increase in the oxygen amount from 11.0 at.% to 20.0 at.%. The maximum oxygen concentration for the PFA-NH_3_/air series was at 800 °C with a gradual decrease in the oxygen concentration as the activation temperature rose. The activation of the PFA-NH_3_ series led to a lower oxygen concentration compared to the PFA-NH_3_/air series. Furthermore, the material reached its maximum oxygen concentration of 16.0 at.% after activation at 900 °C. However, an increase in the temperature from 900 °C to 1000 °C led to a rapid decrease in the oxygen concentration from 16.0 at.% to 3.0 at.%.

In the PFA-N_2_ series, the concentration of oxygen decreased with the increasing temperature. At 1000 °C activation, the material’s chemical composition had an oxygen concentration of 2.0 at.%. Nitrogen functional groups were not present in the chemical composition of the PFA-N_2_ series.

The dependencies presented in Figure 1 show that the reaction of the carbon gasification rate is increased by nitrogen doping. This is why the oxygen concentration increases initially for nitrogen-doped materials. However, a similar effect with a lower oxygen content explains the decrease in the oxygen concentration with temperature for the PFA-N_2_ series.

**Figure 2:**
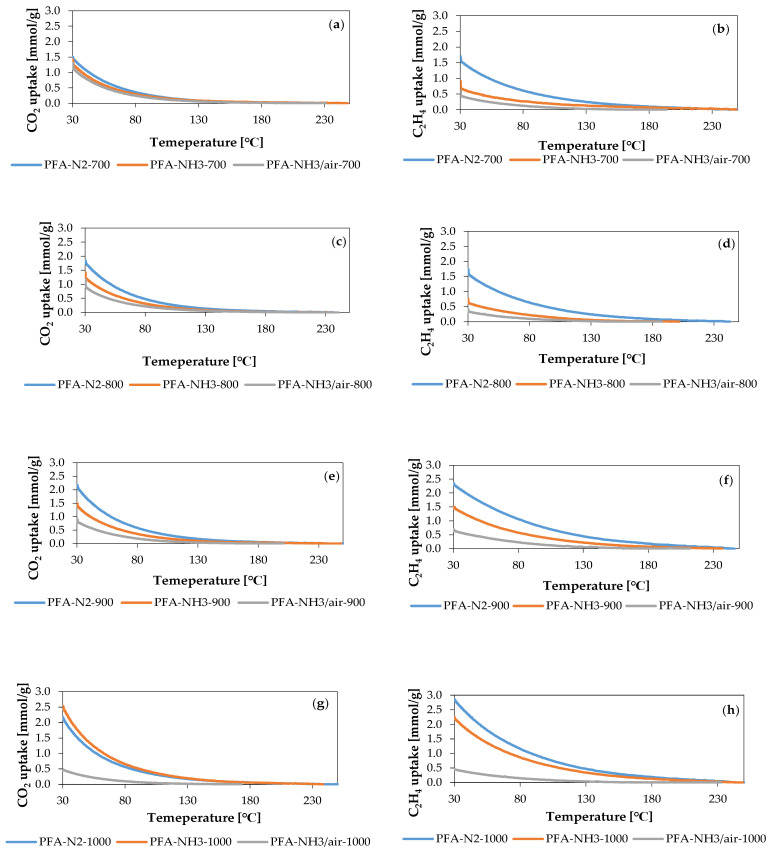
The CO_2_ and C_2_H_4_ uptake for activated carbons activated at (**a**,**b**) 700 °C; (**c**,**d**) 800 °C; (**e**,**f**) 900 °C; and (**g**,**h**) 1000 °C.

At a temperature of 30 °C, we measured the uptake of CO_2_ and C_2_H_4_ using the TGA technique. Figure 2 displays the TGA curves.

The PFA-N_2_ series materials exhibit the highest sorption capacities for carbon dioxide and ethylene. As the temperature of activation increases, the sorption of both gases also increases. Upon comparing their sorption capacities, it is evident that ethylene is adsorbed more than carbon dioxide. Based on the TGA curves, it is evident that ethylene starts to become adsorbed at a higher temperature in the measuring system than carbon dioxide. Ethylene is adsorbed at 180 °C, while carbon dioxide is adsorbed at 130 °C.

The PFA-NH_3_ series and PFA-NH_3_/air series showed lower uptakes of both carbon dioxide and ethylene compared to the N_2_ series.

Carbon dioxide uptake insignificantly increased with an increase in the activation temperature in the PFA-NH_3_ series. In contrast, ethylene uptake significantly increased from 0.9 mmol/g to 2.2 mmol/g as the activation temperature increased from 700 °C to 1000 °C.

The PFA-NH_3_/air series showed a decrease in carbon dioxide uptake and a stable ethylene uptake with the increase in the activation temperature. The uptake of carbon dioxide decreased from 1.3 mmol/g to 0.5 mmol/g as the temperature increased. Ethylene adsorption varied between 0.4 mmol/g and 0.7 mmol/g with an increasing activation temperature.

Table 4 presents a comparison of carbon dioxide uptake by activated carbons derived from various raw materials. The comparison takes into account the raw material, nitrogen source, and activation method.

The data in Table 4 suggest that activated carbons modified with nitrogen compounds have higher CO_2_ uptakes than those without nitrogen atoms in their chemical composition (e.g., PFA). It is important to note that the raw material structure also affects the formation of the porous structure. Plant-derived raw materials have structures consisting of conductive tissue, which forms the primary porous structure of carbonaceous materials. It is important to note that PFA has a solid structure and does not contain any tubules. As a result, activated carbons obtained from natural raw materials will have a more developed porous structure than those obtained from certain polymers. However, modifying activated carbons with nitrogen compounds while simultaneously activating them with KOH is a common practice known to strongly develop the porous structure. It is challenging to determine which element, nitrogen or potassium, is directly responsible for the development of the porous structure. In our experiment, we activated the carbons with CO_2_ to eliminate the effect of potassium. This allowed us to investigate the influences of nitrogen- and oxygen-containing functional groups on both the sorption capacity and the development of the porous structure. The results show that activated carbons produced in an ammonia atmosphere or an ammonia/air mixture have lower CO_2_ sorption capacities than those produced in a nitrogen atmosphere.

In Table 5, the comparison of ethylene uptake by various activated carbons is presented.

Table 5 shows that the carbon materials obtained (PFA-N_2_ series, PFA-NH_3_ series) achieved ethylene uptake comparable to those reported in the literature for activated carbons. The only exception to this is activated carbon with the PFA-NH_3_/air series. The material’s ability to adsorb ethylene differs significantly from what is reported in the literature.

**Figure 3:**
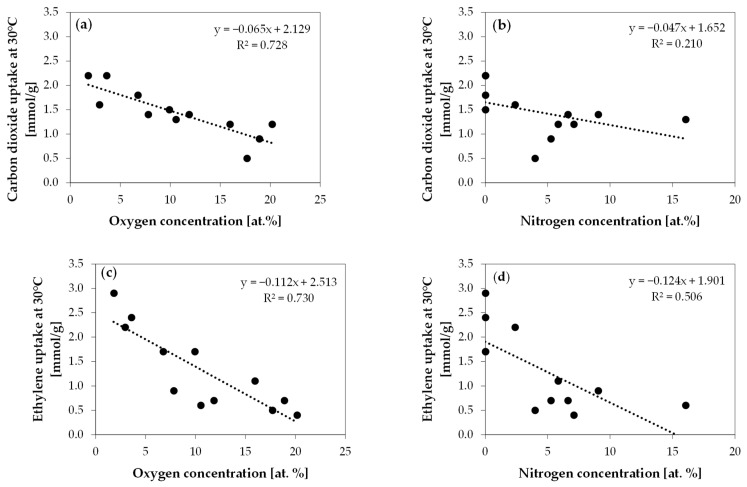
Correlation between (**a**) oxygen concentration and carbon dioxide uptake, (**b**) nitrogen concentration and carbon dioxide uptake, (**c**) oxygen concentration and ethylene uptake, and (**d**) nitrogen concentration and ethylene uptake.

The correlation between the concentrations of oxygen and nitrogen atoms and the adsorption of carbon dioxide and ethylene is shown in Figure 3.

Based on Figure 3, it can be noticed that the oxygen concentration is negatively correlated with the uptake of both carbon dioxide and ethylene. The analogous correlation with the nitrogen concentration is much lower. In both instances, an increase in the concentration of oxygen atoms on the surfaces of activated carbons is correlated with the decrease in gas adsorption. The correlation of the content of nitrogen functional groups with carbon dioxide uptake is negligible. In the case of ethylene uptake, one can observe a correlation and conclude that nitrogen functional groups decrease ethylene uptake.

Both the oxygen and nitrogen functional groups can appear in different forms. For this reason, the impact of individual oxygen and nitrogen functional groups on the adsorption of carbon dioxide and ethylene was investigated. The functional groups containing oxygen and nitrogen were identified using XPS signals of O1s and N1s.

Based on the deconvolution of oxygen signals, the binding energies for particular functional groups were as follows: C=O (530.8 ± 0.3), C-O (532.2 ± 0.3), COOH (533.9 ± 0.3), N=O (534.8 ± 0.3), and H_2_O (536.4 ± 0.3). The O1s signal’s deconvolution can be found in the Supplementary Materials (Figure S3).

**Figure 4:**
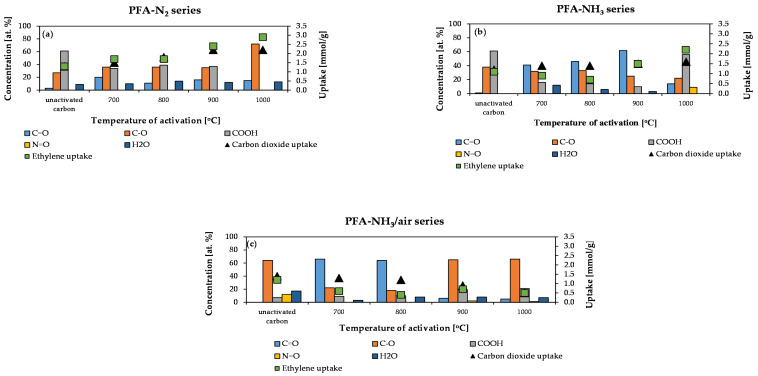
Sorption performance and the concentration of oxygen functional groups on the surfaces of (**a**) PFA-N_2_ series, (**b**) PFA-NH_3_ series, and (**c**) PFA-NH_3_/air series materials.

Figure 4 provides a comparison between the concentration of oxygen-containing functional groups and the uptakes of carbon dioxide and ethylene in the obtained materials.

In the PFA-N_2_ series, it is apparent that the concentration of COOH functional groups decreases as the activation temperature increases. Furthermore, the concentration of C-O groups increases with an increasing activation temperature. It should be noted that a significant increase in the concentration of C-O groups occurs at 1000 °C. The concentration of C=O groups is comparable for each activation temperature.

In the PFA-NH_3_ series, the concentration of COOH groups gradually decreases as the activation temperature is raised from 700 °C to 900 °C before sharply increasing at 1000 °C. Furthermore, it is noted that the concentration of C=O groups increases as the activation temperature rises from 700 °C to 900 °C before abruptly decreasing at 1000 °C. Figure 3b illustrates the correlation between the concentration of C=O groups and the sorption capacity for carbon dioxide. It is shown that as the concentration of C=O groups increases, the sorption capacity for carbon dioxide decreases.

In the PFA-NH_3_/air series, the concentration of COOH groups rises with the increase in the activation temperature. Furthermore, it has been observed that the adsorption of carbon dioxide and ethylene decreases with an increasing activation temperature. At 700 °C and 800 °C, there is a high concentration of C=O groups and a low concentration of C-O groups. At 900 °C, a rapid decrease in the concentration of C=O groups can be observed, accompanied by an increase in the concentration of C-O groups.

The sorption capacity of unactivated carbons is primarily determined by their porous structures with limited influence from functional groups. Poorly developed pores hinder the transfer of gas molecules to adsorption sites, thereby limiting their impacts on adsorption.

**Figure 5:**
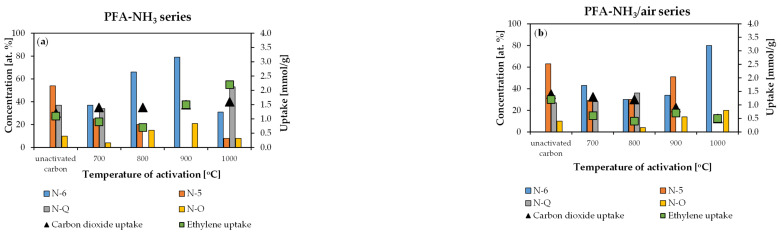
Concentration of nitrogen functional groups on the surfaces of (**a**) PFA-NH_3_ series and (**b**) PFA-NH_3_/air series materials.

Nitrogen groups were determined by the following binding energies: pyridinic N-6 (398.4 ± 0.3), pyrrolic N-5 (399.6 ± 0.3), quaternary N-Q (401.1 ± 0.3), and nitrogen oxides N-O (402.1 ± 0.3). The results are presented in Figure 5. The N1s signal’s deconvolution can be found in the Supplementary Materials (Figure S4).

The N1s signals were deconvoluted to determine the concentrations of nitrogen functional groups in the obtained materials. Pyridynic nitrogen (N-6) dominated in the PFA-NH_3_ series, increasing from 700 °C to 900 °C but decreasing at 1000 °C. There were smaller amounts of pyrrolic nitrogen (N-5) and nitrogen oxide (N-O) groups present. The former decreased with an increasing activation temperature, while the latter increased from 700 °C to 900 °C before decreasing sharply at 1000 °C.

In the case of the PFA-NH_3_/air series, the pyridinic nitrogen (N-6) concentration decreased between 700 °C and 900 °C and then rose sharply at 1000 °C. The pyrrolic nitrogen (N-5) content increased from 700 °C to 900 °C, but there was no pyrrolic nitrogen at 1000 °C. Quaternary nitrogen (N-Q) only appeared after activation at 700 °C and 800 °C. The nitrogen oxide (N-O) concentration increased from 800 °C to 1000 °C.

Except for the surface chemical composition, the structural parameters also affect the sorption properties. This is why the specific surface area (SSA) and pore volume were determined based on nitrogen adsorption at −196 °C. The results are presented in Table 6. The nitrogen adsorption isotherms at −196 °C and the pore size distribution can be found in the Supplementary Materials (Figures S5 and S6).

Table 6 shows that the obtained materials have more developed microporous structures than mesoporous structures. The most developed porous structure was found in the N_2_ series with an increase in the micropore volume from 0.02 cm^3^/g to 0.27 cm^3^/g. The micropore volume was slightly lower in the materials produced via carbonization in an ammonia atmosphere than those under a nitrogen atmosphere. However, the PFA-NH_3_-1000 sample exhibited slightly more porous properties than the PFA-N_2_-1000 sample. Materials from the PFA-NH_3_/air series were found to have significantly lower pore volumes and specific areas compared to both the PFA-N_2_ series and PFA-NH_3_ series materials.

When measuring N_2_ adsorption at −196 °C, it can be challenging to determine the volume of narrow micropores due to the requirement of a very low relative pressure. Additionally, diffusion limitations can occur at a low relative pressure and very low temperatures, preventing N_2_ molecules from reaching the narrowest pores. In contrast, CO_2_ molecules have a high saturation pressure at 0 °C, which enables testing within a low relative pressure range of 10^−3^ to 10^−5^ using an instrument that operates without low pressure [82]. Therefore, the volumes of narrow pores (diameter lower than 1.0 nm) were determined based on the carbon dioxide adsorption at 0 °C. It is worth underlining that pores with diameters lower than 1.0 nm are crucial for carbon dioxide and ethylene sorption.

The impact of the carbonization atmosphere on the microporous structure of PFA-derived materials was determined from the CO_2_ adsorption isotherms at 0 °C (Figure S7) and the pore size distribution (Figure S8). The carbon dioxide uptake at 0 °C and pore volumes are provided in Table 7.

The above table contains columns with cumulative pore volumes up to 0.7 nm and up to 0.8 nm, which are responsible for carbon dioxide sorption at 30 °C and 0 °C, respectively. The cumulative pore volume up to 1.0 nm is responsible for ethylene sorption at 30 °C.

The PFA-N_2_-1000 sample achieved the highest level of carbon dioxide uptake at 0 °C (3.9 mmol/g). As the activation temperature increased, carbon dioxide uptake for the PFA-N_2_ series increased from 2.4 mmol/g to 3.9 mmol/g.

It is evident that both the PFA-NH_3_-700 and PFA-NH_3_/air-700 samples have considerably lower carbon dioxide uptakes than the PFA-N_2_-700 sample. In the PFA-NH_3_ series materials, the sorption capabilities for carbon dioxide increased with further activation at higher temperatures. Table 7 indicates that the development of pores in the carbons of the PFA-NH_3_/air series does not occur during subsequent activation processes. The PFA-NH_3_/air series showed a contrasting trend where the carbon dioxide uptake declined as the activation temperature rose.

**Figure 6:**
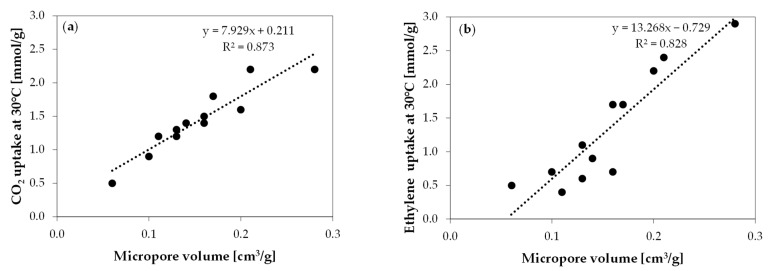
The correlation between micropore volume and (**a**) carbon dioxide uptake at 30 °C and (**b**) ethylene uptake at 30 °C.

Figure 6 provides the correlation between carbon dioxide and ethylene uptakes and micropore volume.

Based on Figure 6, it can be observed that the adsorption of both carbon dioxide and ethylene increases as the micropore volume increases.

We determined the correlation between particular oxygen functional groups and micropore volume based on Figure S9. One can conclude that the concentration of C-O functional groups has a positive correlation with micropore development during activation processes carried out at temperatures of 700 °C and 800 °C. After activation at a temperature of 900 °C, the presence of C-O groups is negligibly correlated with the micropore volume. However, when activated at temperature of 1000 °C, the presence of C-O groups is negatively correlated with the micropore volume. On the contrary, it has been observed that the presence of C=O functional groups negatively correlates with the development of micropores following activation at temperatures of 700 °C and 800 °C. However, no such correlation is observed with micropore volume after activation at temperatures of 900 °C and 1000 °C. Regarding COOH functional groups, it is important to note that their concentration has a positive correlation with the development of micropores after activation at each temperature.

Additionally, based on Figures S10 and S11, we aimed to establish a correlation between particular oxygen groups and carbon dioxide and ethylene uptakes.

Based on Figures S9–S11, it can be noted that, following activation processes carried out at temperatures of 700 °C and 800 °C, high concentrations of C-O and COOH functional groups occur and correlates positively with the adsorption of both carbon dioxide and ethylene. On the contrary, a high concentration of C=O functional groups negatively correlates with carbon dioxide and ethylene uptakes. After being activated at temperatures of 900 °C and 1000 °C, there is no significant correlation between C-O functional groups and carbon dioxide and ethylene uptakes. Additionally, the correlation between the concentration of COOH groups and carbon dioxide and ethylene uptakes is moderate. When activated at a temperature of 900 °C, there is no significant correlation between the uptake of both gases and the concentration of C=O groups. A positive correlation between the high concentration of C=O groups and the adsorption of carbon dioxide and ethylene only occurs after activation at a temperature of 1000 °C.

One must note that the correlation of two parameters is not the same as the impact of one on the other. For example, a high positive correlation between C-O group concentration and CO_2_ uptake may only indicate that such functional groups positively affect CO_2_ sorption. However, it is also possible that other parameters affect both the C-O group concentration and CO_2_ sorption. Hence, a positive relationship might be apparent.

The influence of heteroatoms on the sorption properties of carbonaceous materials is hard to determine because sorption uptakes are dependent on many parameters such as the micropore volume and chemistry of the surface, i.e., the surface elemental concentration and kind of functional group containing heteroatom. In the process of activated carbon preparation, these parameters vary and affect each other. As a result, it is virtually impossible to vary one parameter while keeping the others constant. Thus, determining the influence of a given parameter on sorption is difficult. In the case of commercial activated carbons, there are additional parameters, i.e., the inorganic matter content and ash content, which make the investigation even more difficult [83].

In order to pinpoint the problem, the sorption capacities were normalized and compared to crucial pore volumes. This means that the sorption capacity, expressed in mmol/g, was divided by the crucial pore volumes in Table 7. The crucial pore volumes for CO_2_ and C_2_H_4_ were determined in reference [45]. The results are presented in Table 8.

Normalization allows for the difference in material sorption properties caused by different chemical properties of the surface to be determined. The analysis of the PFA-N_2_ series results shows the impact of oxygen doping. The highest values of the normalized sorption properties of CO_2_ correspond to the highest oxygen content. The higher differences are observed for sorption capacities at 30 °C (pores 0.7 nm) compared to 0 °C. In the case of CO_2_ sorption properties, the effect is smaller but still visible. In the case of C_2_H_4_ sorption at 30 °C, the presence of oxygen inhibits sorption, and the highest normalized sorption capacities were observed for the sample with the lowest oxygen content.

Analyzing the PFA-NH_3_ and PFA-NH_3_/air series is more problematic as these samples contain both oxygen and nitrogen in different ratios. One can notice that in both series, the high normalized sorption capacities of C_2_H_4_ correspond to a low concentration of nitrogen. Therefore, one can claim that the nitrogen content inhibits C_2_H_4_ sorption. However, the impact of nitrogen doping on CO_2_ adsorption is not straightforward. The highest normalized CO_2_ sorption values for the PFA-NH_3_ series activated at 800 °C and 900 °C could be caused by a high oxygen content. However, even higher oxygen contents present in the PFA-NH_3_/air series resulted in lower values of this parameter. It is not clear whether there is an optimal oxygen concentration for the highest CO_2_ sorption properties or whether the type of oxygen–carbon group plays a role. One can claim that the impact of nitrogen on CO_2_ sorption properties is negligible as the samples with very high and very low nitrogen concentrations exhibit close-to-average sorption properties. However, there were no samples with high nitrogen contents and low oxygen contents to strengthen this claim.

The normalized sorption capacities in Table 8 can be compared with the densities of liquid CO_2_, which are 0.91 and 0.60 g/cm^3^ for 0 °C and 30 °C, respectively [84]. These values, expressed in mmol/(g×cm^3^), are 20.7 and 13.6, respectively. When the value of CO_2_ adsorption in Table 4 is greater than the density of liquid CO_2_, one can conclude that surface chemistry has a positive impact on sorption. Conversely, the lower values denote a negative impact.

**Figure 7:**
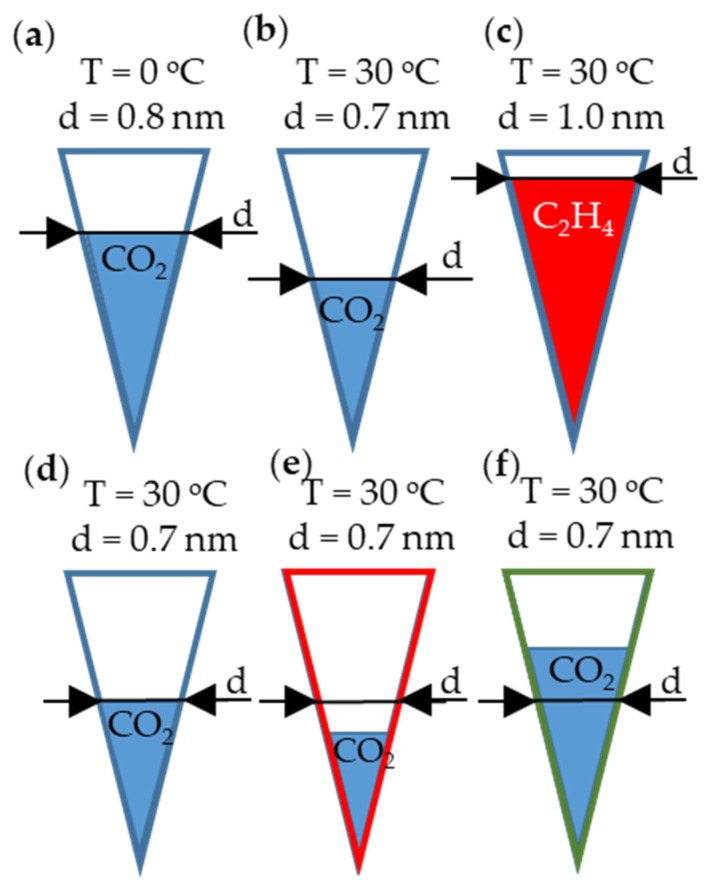
The model of capillary condensation of gases. The crucial pore diameter for the sorption of a given gas is denoted as “d”. (**a**) CO_2_ condensation at 0 °C; (**b**) CO_2_ condensation at 30 °C; (**c**) ethylene condensation at 30 °C; (**d**) condensation without heteroatoms’ impact; (**e**) condensation with negative impact of surface chemistry; (**f**) condensation with positive impact of surface chemistry.

The idea that the surface chemistry has an impact on the sorption of the gases is presented in Figure 7.

One can notice that, at a higher temperature, a lower crucial pore diameter is required for capillary condensation to occur (Figure 7a,b). The capillary condensation of ethylene occurs at a greater pore diameter compared to CO_2_ (Figure 7c). Depending on chemistry of the surface, there may not be an impact on capillary condensation (blue line) (Figure 7d), or this impact can be negative (red line) or positive (green line) (Figure 7e,f, respectively).

**Figure 8:**
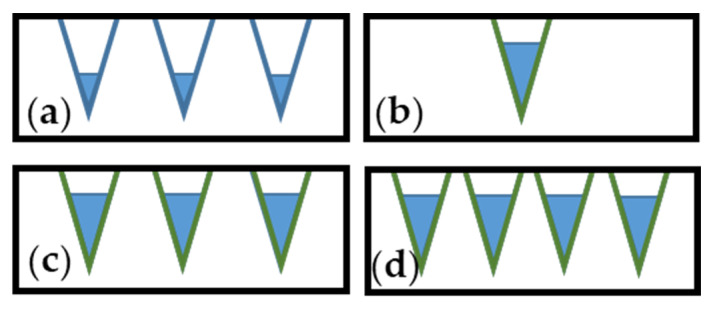
The model of capillary condensation in materials of different porosities and surface chemistries. (**a**) Moderate porosity and neutral surface chemistry impact; (**b**) low porosity and high surface chemistry impact; (**c**) moderate porosity and high surface chemistry impact; (**d**) high porosity and high surface chemistry impact.

The model presented in Figure 7 shows that a high volume of crucial pores is detrimental for high gas uptake. When the volume of pores is greater than the crucial pore diameter, this has a negligible impact on gas uptake. The chemistry of the surface can impact gas uptake; however, a high volume of crucial pores is indispensable. This idea is presented in Figure 8.

The materials with high porosity, e.g., PFA-N2-1000, and high surface chemistry impact, e.g., PFA-NH_3_-900, were obtained (Figure 8a,b, respectively). However, it is still a challenge to obtain materials with high or even higher porosity and high positive impacts on the surface chemistry (Figure 8c,d, respectively).

To conclude, it is difficult to explain the direct impacts of specific carbon–nitrogen or carbon–oxygen surface groups on the sorption properties of CO_2_ or C_2_H_4_. However, one can find at which activation temperature a given surface group is dominant (cf. Figure 4 and Figure 5).

## 3. Materials and Methods

### 3.1. Materials

#### 3.1.1. Preparation of Polyfurfuryl Alcohol

An amount of 60.0 mL of furfuryl alcohol (Merck, Darmstadt, Germany; CAS:98-00-0) was mixed with 8.0 g of maleic acid (Chempur, Piekary Śląskie, Poland; CAS: 110-16-7) on a magnetic stirrer for 30 min at ambient temperature. The solution was then placed in an oven and heated for 10 min at 40 °C, 50 °C, and 80 °C and for 18 h at 200 °C. The cross-linked polyfurfuryl alcohol was ground in a laboratory mill.

#### 3.1.2. Carbonization of Polyfurfuryl Alcohol

An amount of 50.0 g of crushed polyfurfuryl alcohol was placed in a ceramic boat and put into a tube furnace (STF 15/180, Carbolite Gero, Derbyshire, UK). The sample was heated to 600 °C and held for 4 h before cooling to room temperature. The temperature ramp was set at 5 °C/min. The carbonization process was carried out in three distinct atmospheres: nitrogen, ammonia (amination), and a mixture of ammonia and air (ammoxidation). For nitrogen and ammonia, the gas flow was set at 50 mL/min. For ammoxidation, the total flow of ammonia and air was 50 mL/min. The ratio of ammonia to air was set at 3/2. Samples are called PFA-X, where PFA is polyfurfuryl alcohol and X stands for the process atmosphere, e.g., PFA-N_2_, PFA-NH_3_, or PFA-NH_3_/air.

#### 3.1.3. Activation Process

**Figure 9:**
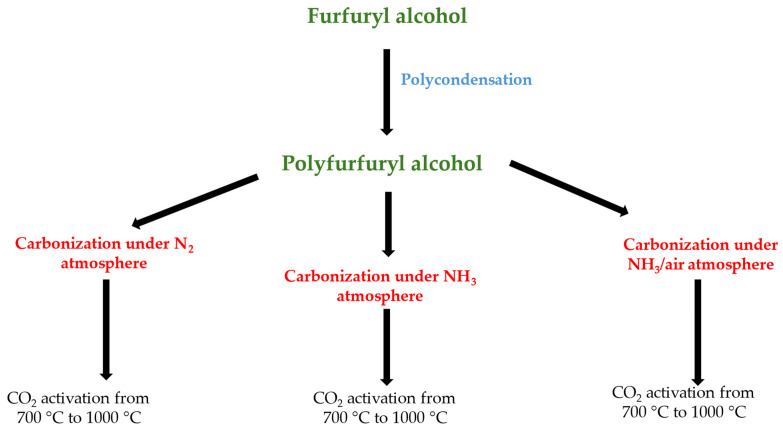
The scheme of obtaining activated carbons from PFA.

An amount of 1.0 g of the carbonized material was placed in a ceramic boat and then placed in a tube furnace (STF 15/180, Carbolite Gero, Derbyshire, UK). The activation process was carried out for 1 h under a carbon dioxide atmosphere in the temperature range of 700 °C to 1000 °C. The carbon dioxide flow was set at 50 mL/min. The temperature ramp was set at 5 °C/min. Samples after activation are named based on the PFA-X-Y scheme, where X represents the atmosphere of carbonization and Y stands for the temperature of activation. The scheme of obtaining activated carbons from PFA was provided on Figure 9. 

### 3.2. Methods

X-ray photoelectron spectroscopy (XPS) was used to determine the surface chemical composition of the carbonaceous materials produced (PREVAC, Rogów, Poland). Data were analyzed using the CasaXPS 2.3.16 software.

Sorption capacities of furfuryl-alcohol-based carbons were determined using thermobalance (TGA) operating under 1025 hPa (handmade thermobalance). Carbon dioxide and ethene were used as adsorbates. During measurements, 0.4 g of material was heated under a carbon dioxide or ethene atmosphere to 250 °C and cooled to 30 °C after 10 min. The flow of the gases was set at 40.0 mL/g.

Pore volumes (V_total_, V_micro_, and V_meso_) were determined based on the nitrogen adsorption/desorption isotherms at −196 °C using the QSDFT model (Autosorb, Quantachrome; Boynton Beach, FL, USA). Specific surface areas were estimated from the BET equation.

Based on the carbon dioxide adsorption at 0 °C, the volume of micropores with diameters lower than 1.0 nm were determined using the NLDFT model (Autosorb, Quantachrome). The porosity data were calculated using the QuadraWin 6.0 software.

The surfaces of the precursor and carbons after carbonization processes were imaged using scanning electron microscopy (Hitachi, Tokyo, Japan).

## 4. Conclusions

In conclusion, the ammoxidation process is more efficient than amination for nitrogen doping. It is recommended to use ammoxidation for improved nitrogen doping efficiency. Carbonized materials obtained by ammoxidation contain approximately 25.0 at.% of nitrogen compared to amination, which only contains only 6.0 at.%.

The main factor that impacts the sorption capacities of CO_2_ and C_2_H_4_ is the specific cumulative volume of pores thar are crucial for the sorption of a given gas. However, this study shows that oxygen over a carbon surface enhances the sorption of CO_2_ and inhibits the sorption of C_2_H_4_. In the case of nitrogen, one can firmly claim that it inhibits sorption of C_2_H_4_. The impact of nitrogen on CO_2_ sorption is negligible; however, in this study, it was not possible to separate the impacts of nitrogen and oxygen.

The presence of nitrogen on the surface of carbonaceous precursors promotes selective gasification during the physical activation of carbon. As a result, the content of nitrogen decreases during the activation process.

The kind of carbonization atmosphere has an impact on the initial contents of both oxygen and nitrogen in carbon. This has a further impact on pore development during physical activation in a CO_2_ atmosphere. The highest cumulative pore volumes that are crucial for CO_2_ and C_2_H_4_ adsorption were obtained for carbonization in a nitrogen atmosphere. Amination and ammoxidation led to inferior results.

On the other hand, it was observed that the pore volumes, which are crucial for the sorption of carbon dioxide and ethylene, decrease with an increasing oxygen concentration, leading to a decrease in the sorption capacity for these gases.

## Figures and Tables

**Table 1:** The yields of carbonization under nitrogen, ammonia, and ammonia/air atmospheres.

Atmosphere of Carbonization	Yield of Carbonization [%]
Nitrogen	55
Ammonia	63
Ammonia/air	69

**Table 2:** Surface chemical composition of polyfurfuryl alcohol and carbonized materials at 600 °C.

Sample	Concentration [at.%]		
	C	O	N
PFA	76	24	0
PFA-N_2_	95	5	0
PFA-NH_3_	91	3	6
PFA-NH_3_/air	65	10	25

**Table 3:** The yields of CO_2_ activation processes.

Temperature of Activation [°C]	Yield of Activation Process [%]		
	PFA-N_2_ Series	PFA-NH_3_ Series	PFA-NH_3_/Air Series
700	95	97	96
800	94	94	94
900	83	79	79
1000	70	62	55

**Table 4:** Comparison of CO_2_ uptake by various activated carbons.

Raw Material	Nitrogen Source	CO_2_ Uptake [mmol/g]	Method of Activation	Reference
Macadamia nutshell	Melamine	4.4 at 0 °C	KOH activation	[71]
Coconut shell	-	4.2 at 25 °C	KOH activation	[72]
Sugarcane bagasse	Urea	4.8 at 25 °C	KOH activation	[73]
Coconut shell	Urea	3.7 at 25 °C	K_2_CO_3_ activation	[74]
Chitosan and glucose	Ammonia, chitosan	5.5 at 25 °C	Autoclave	[75]
PPy	-	3.7 at 25 °C	NaOH activation	[76]
Rice husk	Chitosan	3.7 at 25 °C	KOH activation	[77]
PFA	Ammonia nitrate	2.1 at 30 °C	CO_2_ activation	[70]
PFA	-	2.2 at 30 °C	CO_2_ activation	This study
	Ammonia	1.6 at 30 °C		
	Ammonia/air	0.5 at 30 °C		

**Table 5:** Comparison of C_2_H_4_ uptake by various activated carbons.

Raw Material	Nitrogen Source	C_2_H_4_ Uptake [mmol/g]	Reference
Commercial carbon	-	3.1 at 40 °C	[78]
Date seeds	-	2.9 at 30 °C	[79]
Asphalt	-	7.2 at 25 °C	[80]
Hardwood lignosulfonate powder	-	2.2 at 25 °C	[69]
Coffee beans	-	2.3 at 35 °C	[81]
PFA	Ammonium nitrate	3.4 at 30 °C	[70]
PFA	-	2.9 at 30 °C	This study
	Ammonia	2.2 at 30 °C	
	Ammonia/air	0.5 at 30 °C	

**Table 6:** Specific surface areas and pore volumes of obtained materials.

Sample	SSA ^1^ [m^2^/g]	V_total_^2^ [cm^3^/g]	V_micro_^3^ [cm^3^/g]	V_meso_^4^ [cm^3^/g]
PFA-N_2_-700	75	0.04	0.02	0.01
PFA-N_2_-800	105	0.05	0.04	0.01
PFA-N_2_-900	539	0.19	0.15	0.03
PFA-N_2_-1000	746	0.30	0.27	0.00
PFA-NH_3_-700	14	0.01	0.00	0.01
PFA-NH_3_-800	53	0.03	0.02	0.01
PFA-NH_3_-900	188	0.08	0.07	0.00
PFA-NH_3_-1000	768	0.31	0.29	0.00
PFA-NH_3_/air-700	16	0.01	0.01	0.00
PFA-NH_3_/air-800	32	0.02	0.01	0.00
PFA-NH_3_/air-900	116	0.05	0.04	0.00
PFA-NH_3_/air-1000	125	0.05	0.05	0.00

^1^ SSA—specific surface area, calculated using BET equation. ^2^ V_total_—total volume of pores determined from N_2_ adsorption at −196 °C. ^3^ V_micro_—volume of pores with diameter lower than 2 nm, determined from N_2_ adsorption at −196 °C. ^4^ V_meso_—volume of pores with diameter in the range of 2 nm to 50 nm, estimated from N_2_ adsorption at −196 °C.

**Table 7:** Textural parameters and CO_2_ uptakes of obtained materials.

Sample	CO_2_ Uptake at 0 °C [mmol/g]	S_CO2_ [m^2^/g]	V_total_ [cm^3^/g]	V_0.7nm_ [cm^3^/g]	V_0.8nm_ [cm^3^/g]	V_1.0nm_ [cm^3^/g]
PFA-N_2_-700	2.4	520	0.16	0.11	0.11	0.14
PFA-N_2_-800	2.6	556	0.17	0.12	0.12	0.15
PFA-N_2_-900	3.2	689	0.21	0.15	0.16	0.18
PFA-N_2_-1000	3.9	817	0.28	0.18	0.19	0.23
PFA-NH_3_-700	1.9	401	0.14	0.09	0.10	0.11
PFA-NH_3_-800	2.0	425	0.16	0.09	0.09	0.13
PFA-NH_3_-900	1.6	348	0.13	0.07	0.07	0.10
PFA-NH_3_-1000	2.6	544	0.20	0.11	0.12	0.16
PFA-NH_3_/air-700	1.9	407	0.13	0.09	0.10	0.11
PFA-NH_3_/air-800	1.6	316	0.11	0.05	0.09	0.10
PFA-NH_3_/air-900	1.3	262	0.10	0.05	0.06	0.08
PFA-NH_3_/air-1000	0.8	169	0.06	0.03	0.04	0.05

**Table 8:** Normalized sorption capacities.

Sample	Oxygen Concentration [at.%]	Nitrogen Concentration [at.%]	CO_2_ Uptake, mmol/cm^3^ (d = 0.7 nm)	CO_2_ Uptake, mmol/cm^3^ (d = 0.8 nm)	C_2_H_4_ Uptake, mmol/cm^3^ (d = 1.0 nm)
PFA-N_2_-700	10	0	14	22	12
PFA-N_2_-800	7	0	15	22	11
PFA-N_2_-900	4	0	14	20	13
PFA-N_2_-1000	2	0	12	21	13
PFA-NH_3_-700	8	9	14	19	8
PFA-NH_3_-800	12	7	16	22	5
PFA-NH_3_-900	16	6	17	23	11
PFA-NH_3_-1000	3	2	13	22	14
PFA-NH_3_/air-700	11	16	13	19	5
PFA-NH_3_/air-800	20	7	13	18	4
PFA-NH_3_/air-900	19	5	15	22	9
PFA-NH_3_/air-1000	18	4	13	20	10

## Data Availability

The data presented in this study are available on request from the corresponding author. The data are not publicly available due to [the large size of the files].

## Supplementary Materials

The following supporting information can be downloaded at https://www.mdpi.com/article/10.3390/molecules29050987/s1, Figure S1: The XPS spectra of PFA, PFA-N_2_, PFA-NH_3_ and PFA-NH_3_/air; Figure S2: SEM image of (a) PFA, (b) PFA-N2, (c) PFA-NH3, (d) PFA-NH3/air; Figure S3: O1s peak deconvolution for (a–c) carbons activated at 700 °C, (d–f) carbons activated at 800 °C, (g–i) carbons activated at 900 °C, (j–l) carbons activated at 1000 °C; Figure S4: N1s peak deconvolution for (a,b) carbons activated at 700 °C; (c,d) carbons activated at 800 °C; (e,f) carbons activated at 900 °C; g),h) carbons activated at 1000 °C; Figure S5: Nitrogen adsorption isotherms at −196 °C of (a) PFA-N_2_-series, (b) PFA-NH_3_-series, (c) PFA-NH_3_/air-series; Figure S6: Pore size distribution of (a) PFA-N_2_-series, (b) PFA-NH_3_-series, (c) PFA-NH_3_/air-series calculated from N_2_ adsorption data at −196 °C; Figure S7: CO_2_ adsorption isotherms at 0 °C for (a) PFA-N_2_-series, (b) PFA-NH_3_-series, (c) PFA-NH_3_/air-series; Figure S8: Micropores size distributions based on CO_2_ adsorption at 0 °C for (a) PFA-N_2_-series, (b) PFA-NH_3_-series, (c) PFA-NH_3_/air-series; Figure S9: The correlation between particular oxygen functional groups and micropore volume (a–c) after activation at 700 °C, (d–f) after activation at 800 °C, (g–i) after activation at 900 °C, (j–l) after activation at 1000 °C; Figure S10: The correlation between individual oxygen functional groups and carbon dioxide uptake (a–c) after activation at 700 °C, (d–f) after activation at 800 °C, (g–i) after activation at 900 °C, (j–l) after activation at 1000 °C; Figure S11: The correlation between individual oxygen functional groups and ethylene uptake (a–c) after activation at 700 °C, (d–f) after activation at 800 °C, (g–i) after activation at 900 °C, (j–l) after activation at 1000 °C.
