# Supplementary material for: The Impact of N/O-Functional Groups on the Sorption Capabilities of Activated Carbons Derived from Furfuryl Alcohol
Source: Molecules. 2024 Feb 24;29(5):987. doi: 10.3390/molecules29050987 (PMC10934998; doi:10.3390/molecules29050987)
Supplement: Supplementary file 1 [file molecules-29-00987-s001.zip › molecules-2861701-supplementary.pdf]

Supplementary material for:

The Impact of N/O-Functional Groups on the Sorption Capabilities of Activated Carbons Derived from Furfuryl Alcohol

Agnieszka Kałamaga and Rafał J. Wróbel

Department of Catalytic and Sorbent Materials Engineering, Faculty of Chemical Technology and Engineering, West Pomeranian University of Technology, Piastów 17 Ave., 70-310 Szczecin, Poland

Correspondence: agnieszka.kalamaga@zut.edu.pl (A.K.); rafal.wrobel@zut.edu.pl (R.J.W.)

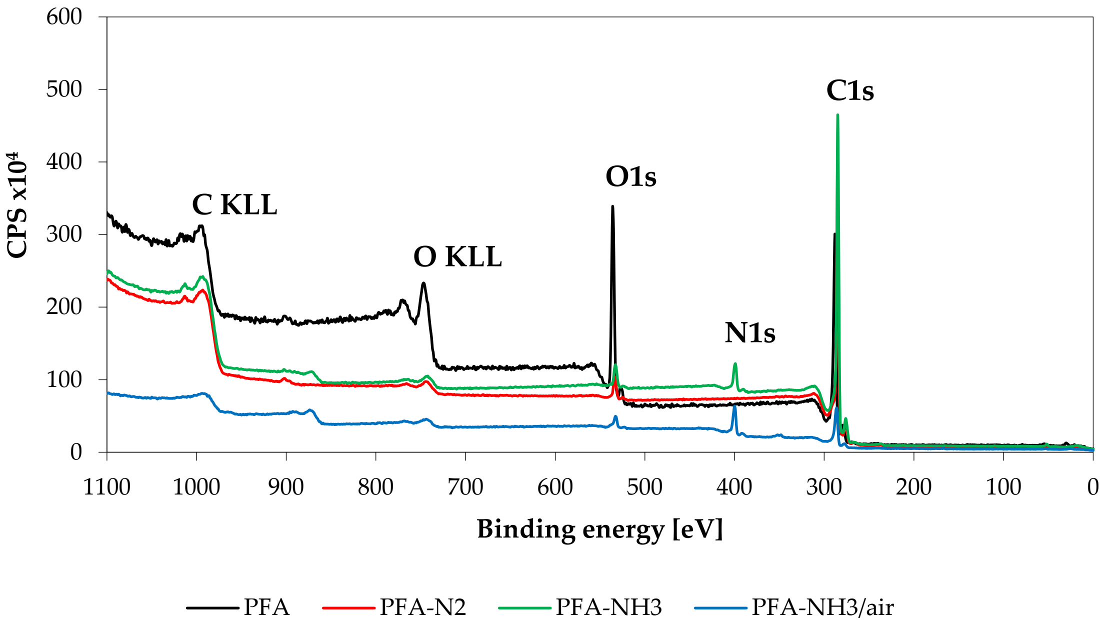

Figure S1. The XPS spectra of PFA, PFA-N_2, PFA-NH_3 and PFA-NH_3/air

a) PFA

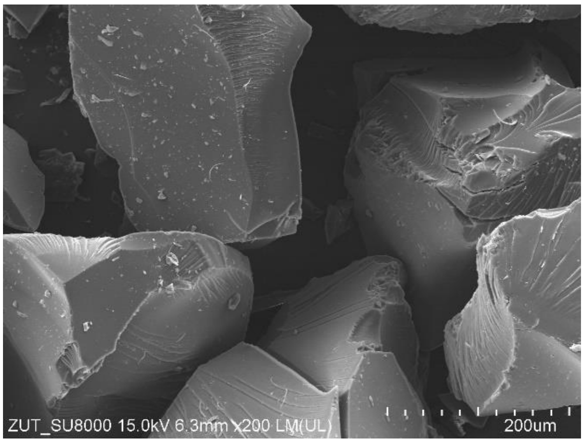

b) PFA-N2

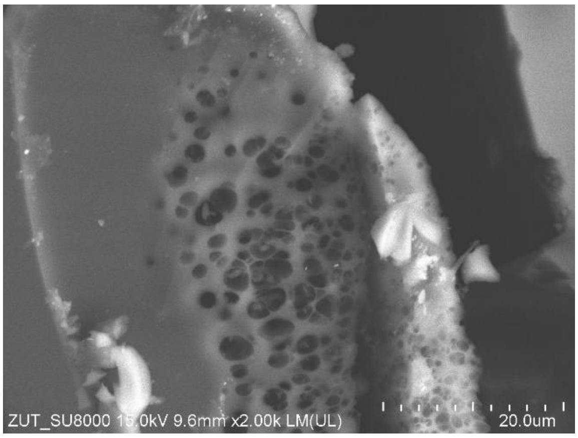

c) PFA-NH3

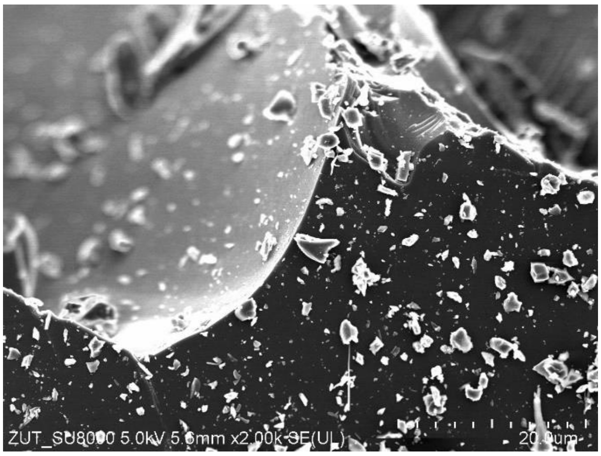

d) PFA-NH3/air

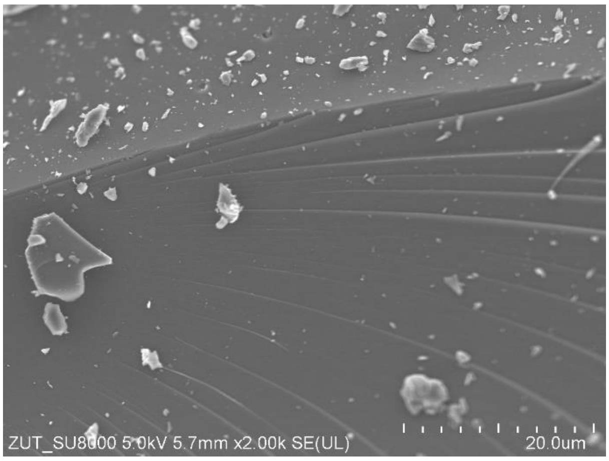

Figure S2. SEM image of a) PFA, b) PFA-N2, c) PFA-NH3, d) PFA-NH3/air

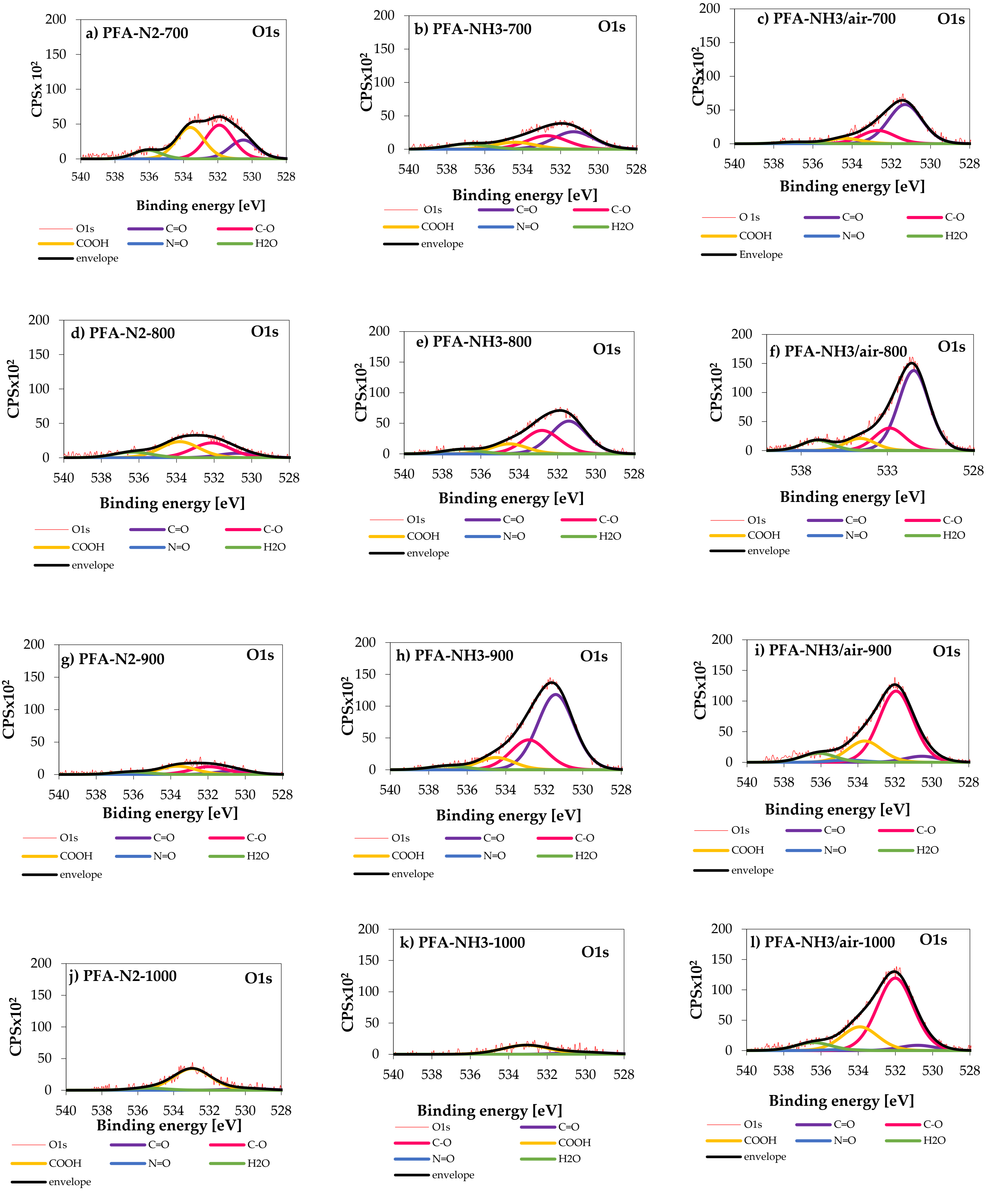
a) PFA-N2-700
O1s
CPSx 10²
Binding energy [eV]
O1s C=O C-O COOH N=O H2O envelope
b) PFA-NH3-700
O1s
CPSx10²
Binding energy [eV]
O1s C=O C-O COOH N=O H2O envelope
c) PFA-NH3/air-700
O1s
CPSx10²
Binding energy [eV]
O 1s C=O C-O COOH N=O H2O Envelope
d) PFA-N2-800
O1s
CPSx10²
Binding energy [eV]
O1s C=O C-O COOH N=O H2O envelope
e) PFA-NH3-800
O1s
CPSx10²
Binding energy [eV]
O1s C=O C-O COOH N=O H2O envelope
f) PFA-NH3/air-800
O1s
CPSx10²
Binding energy [eV]
O1s C=O C-O COOH N=O H2O envelope
g) PFA-N2-900
O1s
CPSx10²
Biding energy [eV]
O1s C=O C-O COOH N=O H2O envelope
h) PFA-NH3-900
O1s
CPSx10²
Binding energy [eV]
O1s C=O C-O COOH N=O H2O envelope
i) PFA-NH3/air-900
O1s
CPSx10²
Binding energy [eV]
O1s C=O C-O COOH N=O H2O envelope
j) PFA-N2-1000
O1s
CPSx10²
Binding energy [eV]
O1s C=O C-O COOH N=O H2O envelope
k) PFA-NH3-1000
O1s
CPSx10²
Binding energy [eV]
O1s C=O C-O COOH N=O H2O envelope
l) PFA-NH3/air-1000
O1s
CPSx10²
Binding energy [eV]
O1s C=O C-O COOH N=O H2O envelope

Figure S3. O1s peak deconvolution for a)-c) carbons activated at 700°C, d)-f) carbons activated at 800°C, g)-i) carbons activated at 900°C, j)-l) carbons activated at 1000°C

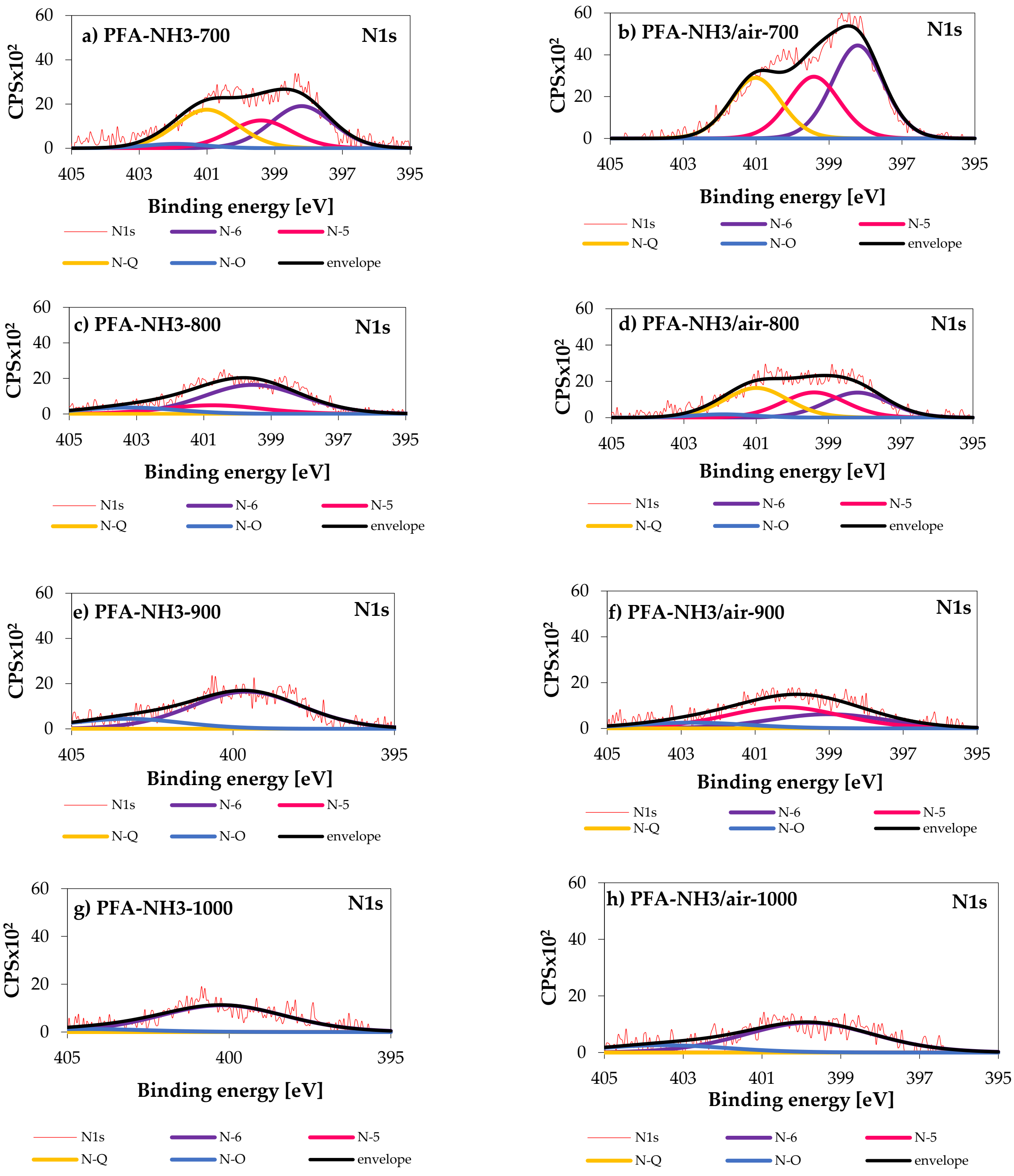

Figure S4. N1s peak deconvolution for a),b) carbons activated at 700°C; c) d) carbons activated at 800°C; e),f) carbons activated at 900°C; g),h) carbons activated at 1000°C

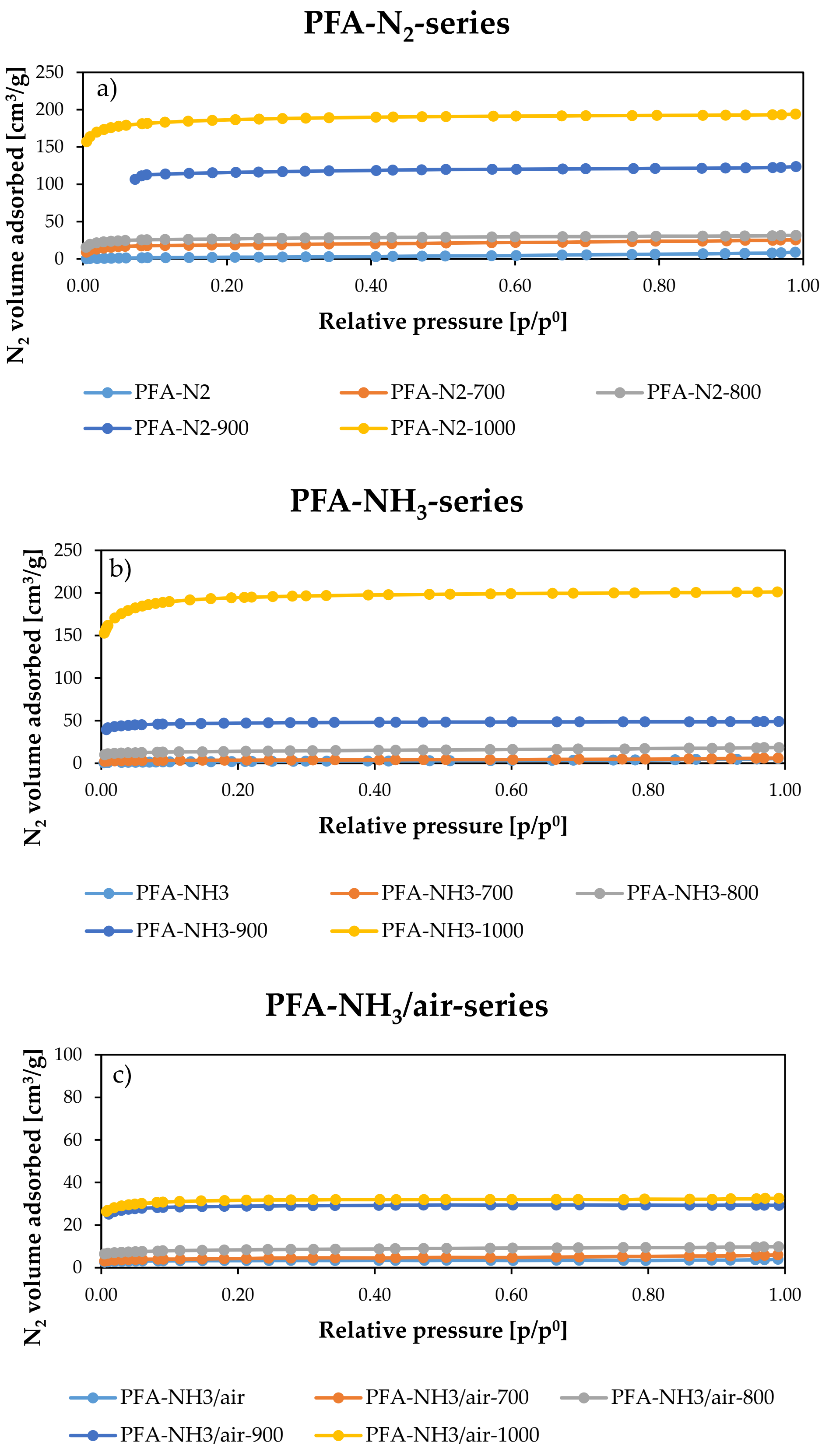

Figure S5. Nitrogen adsorption isotherms at -196°C of a) PFA-N_2-series, b) PFA-NH_3-series, c) PFA-NH_3/air-series

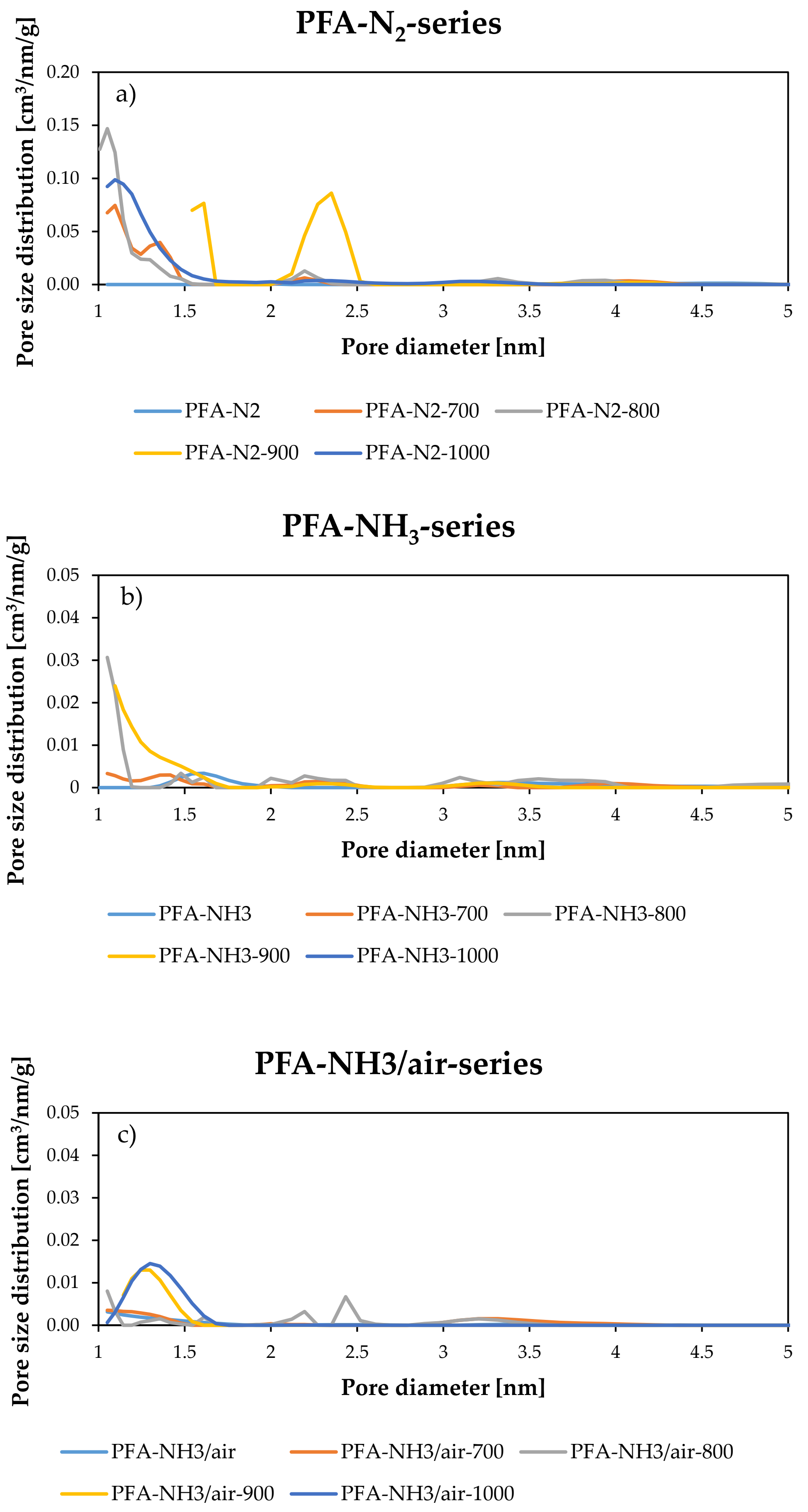

Figure S6. Pore size distribution of a) PFA-N_2-series, b) PFA-NH_3-series, c) PFA-NH_3/air-series calculated from N_2 adsorption data at -196°C

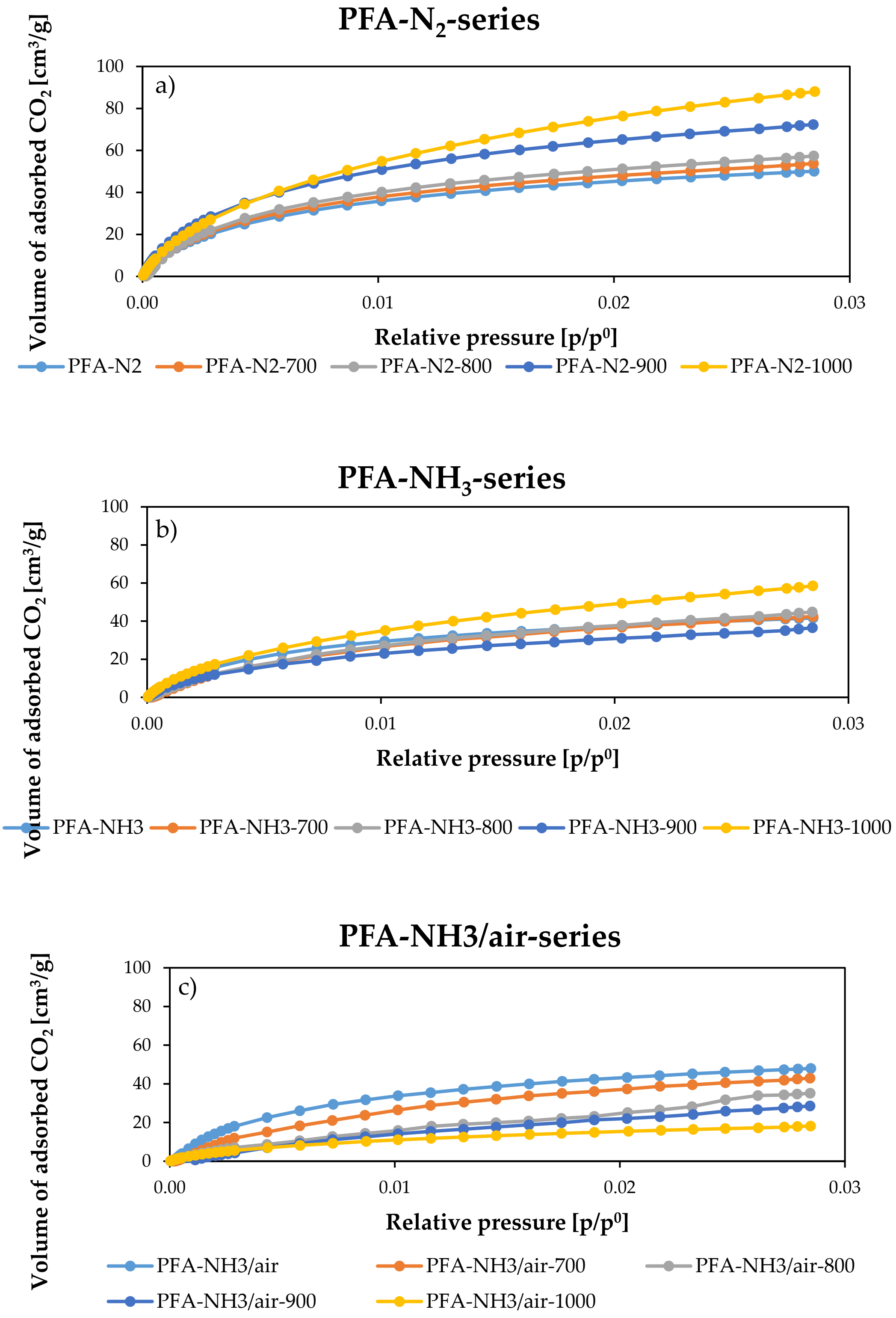

Figure S7. CO_2 adsorption isotherms at 0°C for a) PFA-N_2-series, b) PFA-NH_3-series, c) PFA-NH_3/air-series

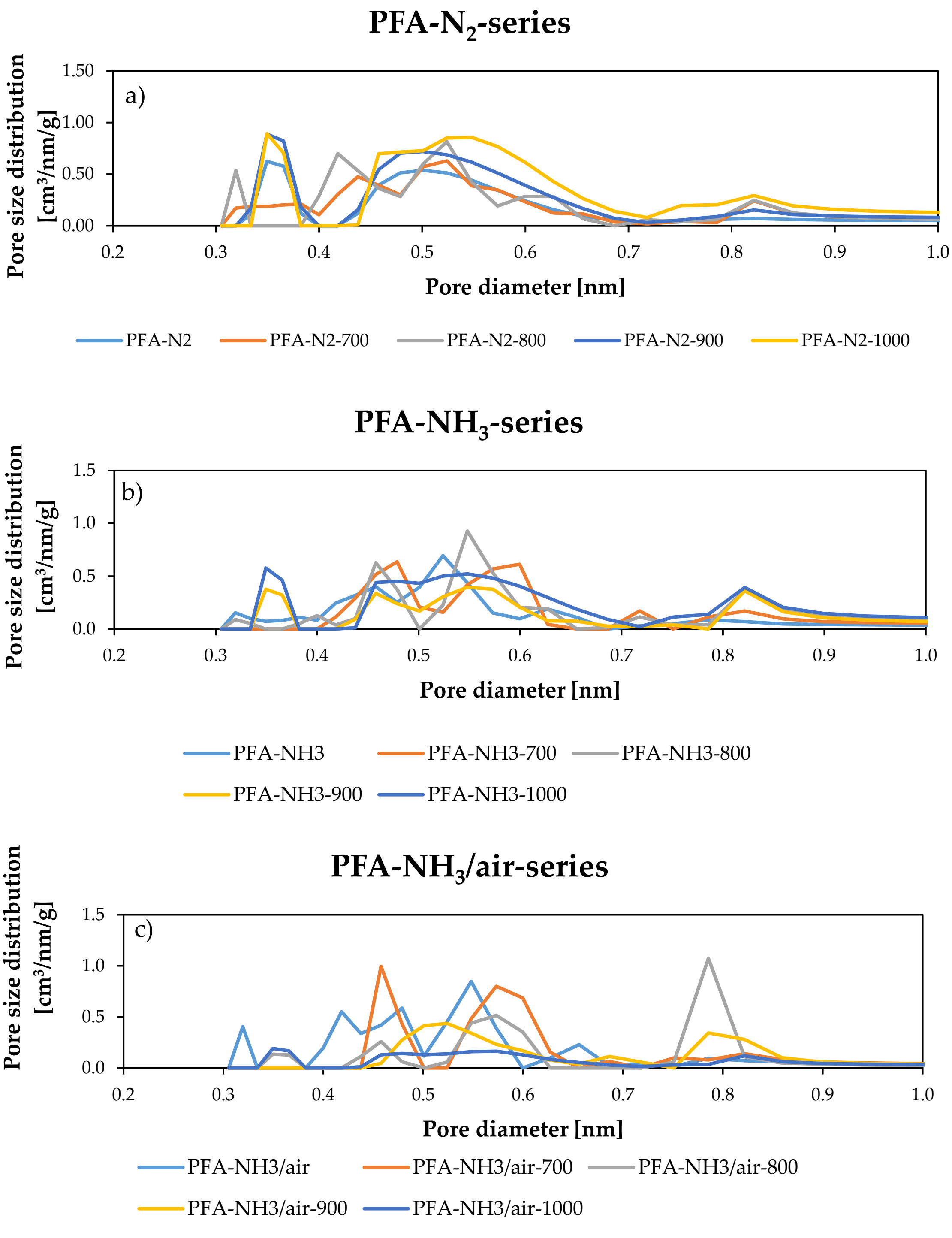

Figure S8. Micropores size distributions based on CO_2 adsorption at 0°C for a) PFA-N_2-series, b) PFA-NH_3-series, c) PFA-NH_3/air-series

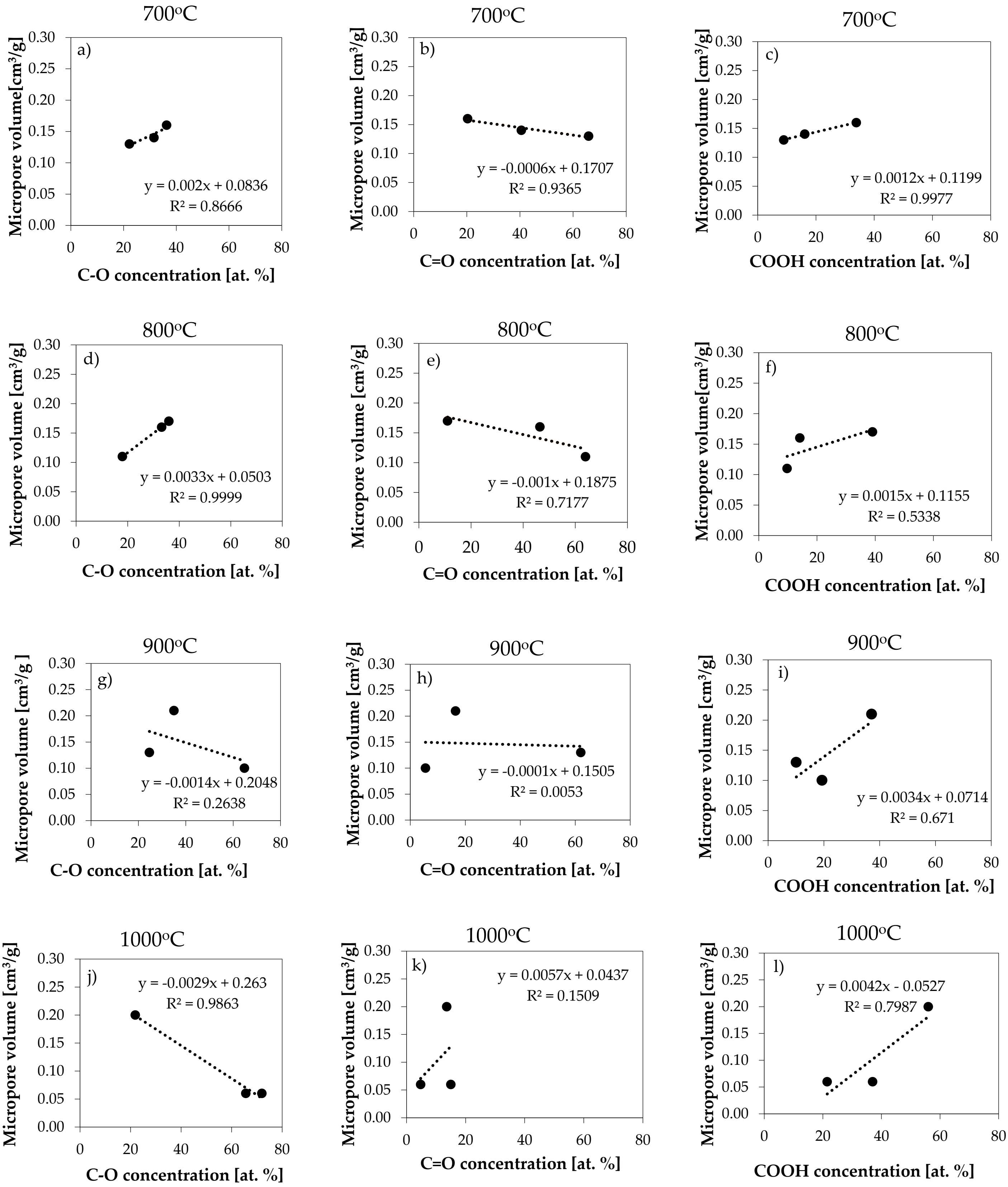

Figure S9. The correlation between particular oxygen functional groups and micropore volume a), b),c) after activation at 700°C, d), e), f) after activation at 800°C, g), h), i) after activation at 900°C, j), k), l) after activation at 1000°C

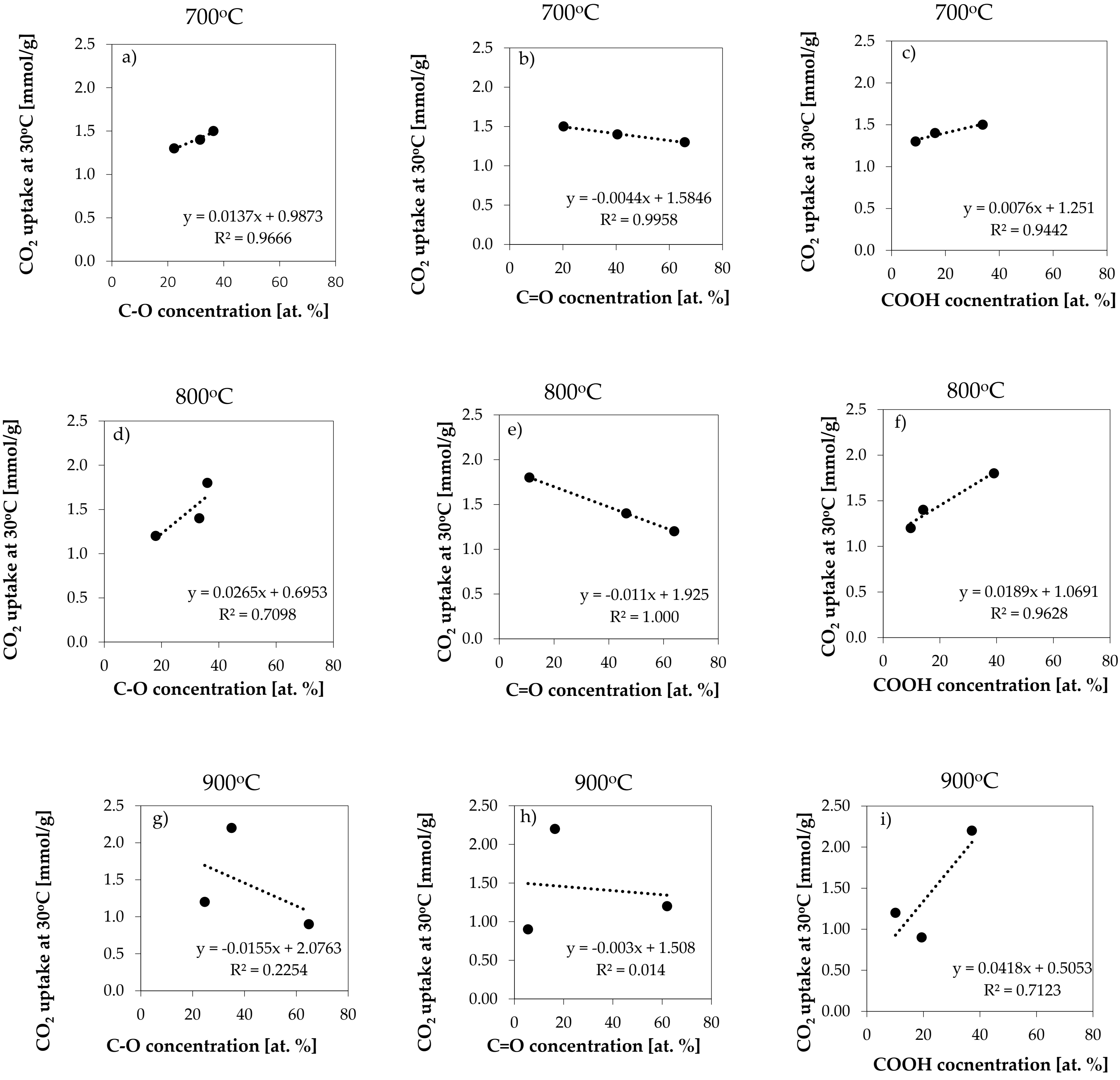

700°C
a)
y = 0.0137x + 0.9873
R² = 0.9666
C-O concentration [at. %]
CO2 uptake at 30°C [mmol/g]
700°C
b)
y = -0.0044x + 1.5846
R² = 0.9958
C=O cocnentration [at. %]
CO2 uptake at 30°C [mmol/g]
700°C
c)
y = 0.0076x + 1.251
R² = 0.9442
COOH cocnentration [at. %]
CO2 uptake at 30°C [mmol/g]
800°C
d)
y = 0.0265x + 0.6953
R² = 0.7098
C-O concentration [at. %]
CO2 uptake at 30°C [mmol/g]
800°C
e)
y = -0.011x + 1.925
R² = 1.000
C=O concentration [at. %]
CO2 uptake at 30°C [mmol/g]
800°C
f)
y = 0.0189x + 1.0691
R² = 0.9628
COOH concentration [at. %]
CO2 uptake at 30°C [mmol/g]
900°C
g)
y = -0.0155x + 2.0763
R² = 0.2254
C-O concentration [at. %]
CO2 uptake at 30°C [mmol/g]
900°C
h)
y = -0.003x + 1.508
R² = 0.014
C=O concentration [at. %]
CO2 uptake at 30°C [mmol/g]
900°C
i)
y = 0.0418x + 0.5053
R² = 0.7123
COOH cocnentration [at. %]
CO2 uptake at 30°C [mmol/g]

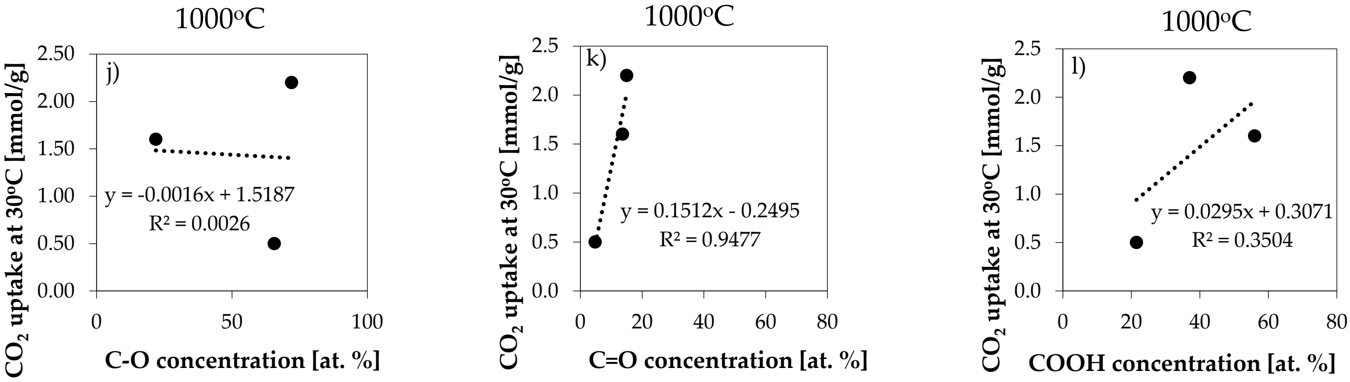

Figure S10. The correlation between individual oxygen functional groups and carbon dioxide uptake a), b), c) after activation at 700°C, d), e), f) after activation at 800°C,g), h),i) after activation at 900°C, j),k),l) after activation at 1000°C,

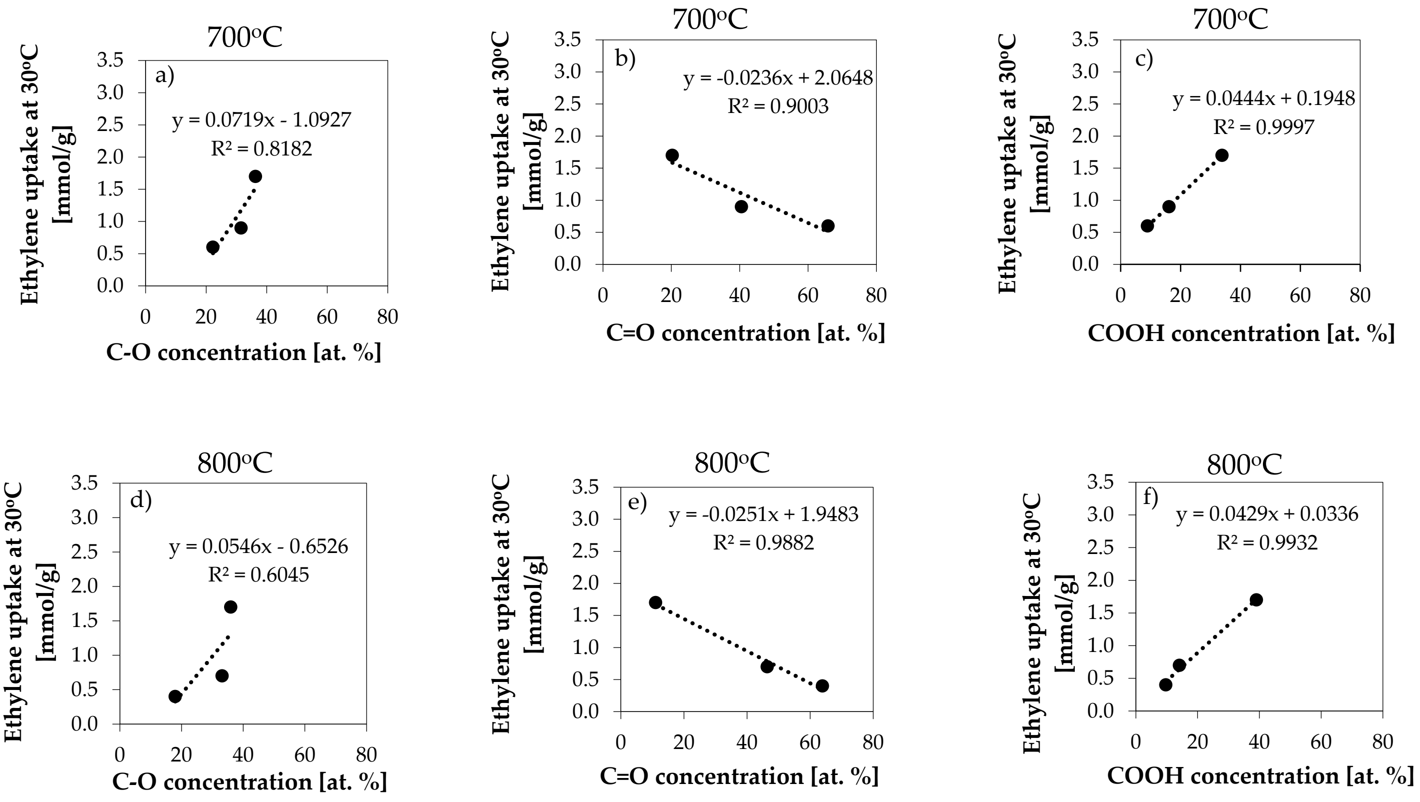

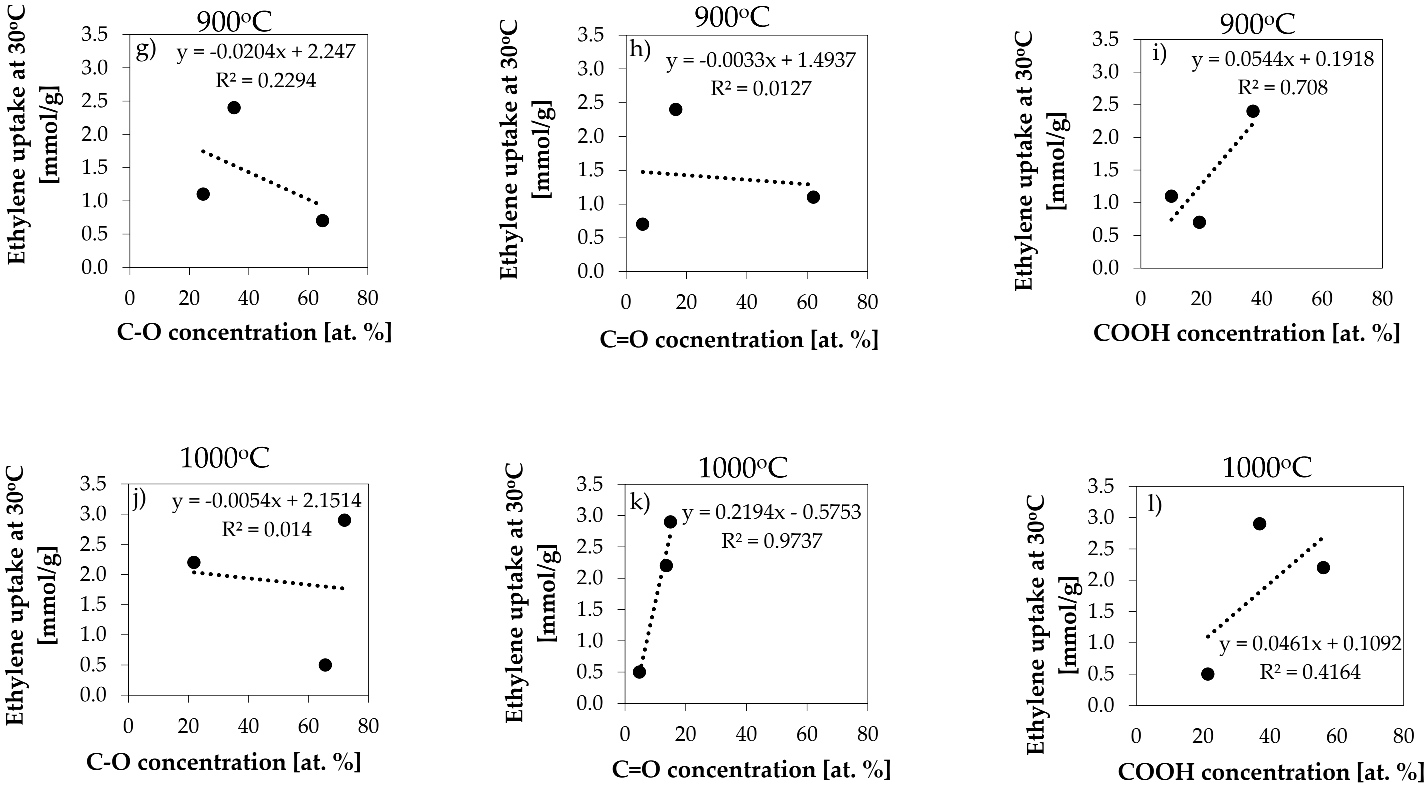

Figure S11. The correlation between individual oxygen functional groups and ethylene uptake a), b),c) after activation at 700°C, d), e), f) after activation at 800°C, g), h), i) after activation at 900°C, j), k), l) after activation at 1000°C
